# Vascular dysfunction caused by loss of Brn-3b/POU4F2 transcription factor in aortic vascular smooth muscle cells is linked to deregulation of calcium signalling pathways

**DOI:** 10.1038/s41419-023-06306-w

**Published:** 2023-11-25

**Authors:** Vaishaali Yogendran, Laura Mele, Oleksandra Prysyazhna, Vishwanie S. Budhram-Mahadeo

**Affiliations:** 1 Molecular Biology Development and Disease, UCL Institute of Cardiovascular Science, London, UK; 2 Clinical Pharmacology Centre, William Harvey Research Institute, Queen Mary University of London, London, UK

**Keywords:** Transcription, Vascular diseases

## Abstract

Phenotypic and functional changes in vascular smooth muscle cells (VSMCs) contribute significantly to cardiovascular diseases (CVD) but factors driving early adverse vascular changes are poorly understood. We report on novel and important roles for the Brn-3b/POU4F2 (Brn-3b) transcription factor (TF) in controlling VSMC integrity and function. Brn-3b protein is expressed in mouse aorta with localisation to VSMCs. Male Brn-3b knock-out (KO) aortas displayed extensive remodelling with increased extracellular matrix (ECM) deposition, elastin fibre disruption and small but consistent narrowing/coarctation in the descending aortas. RNA sequencing analysis showed that these effects were linked to deregulation of genes required for calcium (Ca^2+^) signalling, vascular contractility, sarco-endoplasmic reticulum (S/ER) stress responses and immune function in Brn-3b KO aortas and validation studies confirmed changes in Ca^2+^ signalling genes linked to increased intracellular Ca^2+^ and S/ER Ca^2+^ depletion [e.g. increased, Cacna1d Ca^2+^ channels; ryanodine receptor 2, (RyR2) and phospholamban (PLN) but reduced ATP2a1, encoding SERCA1 pump] and chaperone proteins, Hspb1, HspA8, DnaJa1 linked to increased S/ER stress, which also contributes to contractile dysfunction. Accordingly, vascular rings from Brn-3b KO aortas displayed attenuated contractility in response to KCl or phenylephrine (PE) while Brn-3b KO-derived VSMC displayed abnormal Ca^2+^ signalling following ATP stimulation. This data suggests that Brn-3b target genes are necessary to maintain vascular integrity /contractile function and deregulation upon loss of Brn-3b will contribute to contractile dysfunction linked to CVD.

## Introduction

Cardiovascular diseases (CVD) remain a leading cause of morbidity and mortality, globally [1–3] but the chronic nature of the disease means that early changes that contribute to disease development and progression are still not fully understood. In fact, abnormal vasculature such as reduced arterial compliance and blood vessel (BV) contractility are independent predictors of CVD mortality, particularly in patients with essential hypertension [4–6]. While vascular damage and dysfunction often develop gradually over time, such changes can remain asymptomatic and undetectable at early stages when interventions may be effective for reducing or preventing adverse changes. At later stages, symptoms arising from vascular damage and arterial stiffening are often irreversible and therefore difficult to treat [2, 7].

Therefore, studying the mechanisms that control integrity and function in healthy blood vessels can provide insight into pathophysiological processes that contribute to vascular dysfunction and disease. Large BVs, such as the aorta provide unique opportunities to study vascular dysfunction because such BV’s are highly adapted for sensing pulsatile blood flow and responding rapidly to changes in cardiac output and blood pressure, under different conditions [8]. In the aorta, differentiated vascular smooth muscle cells (VSMCs) in the tunica media (TM), are critical for rapid adaptation to changes in blood by producing elastin (Eln) that confers elastance and recoil [8, 9]. However, VSMCs also produce contractile proteins [e.g. smooth muscle protein 22-alpha (SM22α), alpha smooth muscle actin (α-SMA) and calponin] that regulate vascular tone [10], blood flow and pressure [11, 12], in response to rapid and active changes in intracellular calcium (Ca^2+^), which is, in turn, regulated by the activation or inhibition of Ca^2+^ channels, pumps and other regulatory proteins found in the plasma membrane and intracellular store e.g. sarco-endoplasmic reticulin (S/ER) [13–15]. Therefore, controlling the genes that encode such essential proteins in VSMCs, will be critical for determining vascular function.

This is particularly important because of the inherent plasticity of VSMCs, whereby contractile cells can undergo phenotypic switching in response to injury or chronic stress [e.g. raised intracellular Ca^2+^ or S/ER Ca^2+^depletion and stress; increased reactive oxygen species (ROS), inflammation [16–20]. Under such conditions, VSMCs undergo de-differentiation and switch from contractile cells into mesenchymal phenotypes that acquire migratory, proliferative and secretory properties [9, 14]. Such effects are driven by changes in genes that encode important cellular proteins such as reduction in contractile proteins but increase expression of extracellular matrix (ECM) proteins e.g. collagen that reduces VSMC contractility and contributes to vascular dysfunction such as arterial stiffening and hypertension [12, 14] highlighted.

Transcription factors (TFs) that regulate the rate of transcription of multiple target genes, in a tissue specific manner are critical for determining cell fate [21] and changes in key TFs required for normal vascular integrity and function can lead to adverse vascular changes linked to CVD [22]. The Brn-3b/POUF2 protein (Brn-3b) TF, was identified as a key regulator of gene expression in cardiovascular tissues [23–26]. This TF is characterised by a highly conserved DNA binding POU (pit-oct-unc) homeodomain that can bind to unique BRNF DNA sites in specific gene promoters and either activate or repress gene transcription by RNA polymerase II, depending on cell/tissue type [27–29]. However, Brn-3b can also indirectly regulate gene transcription by binding to and modulating the transcriptional effects of other TFs e.g. p53 tumour suppressor protein and oestrogen receptor (ER) on distinct target genes [24, 25, 28, 30]. Interestingly, the gene encoding Brn-3b consisting of 2 exons and can give rise to 2 distinct isoforms, thought to arise from alternative promoter usage, with the longer Brn-3b (l) protein (43–46 kDa) encoded by exon 1 and exon 2 while the shorter Brn-3b(s) isoform encoded entirely by exon 2 [30–32]. While the precise function of the different Brn-3b isoforms remain unclear, experimental evidence support auto-regulation and positive feedback loop whereby different isoforms can be regulated by each other depending on the tissue of expression [30–33].

Although isolated and widely studied in neuronal cells, Brn-3b is also found in other tissues including cardiovascular system (cardiomyocytes/heart), metabolic tissue (skeletal muscle; adipose tissue), testes and immune cells [peripheral blood mononuclear cells (PBMC), T-cells and monocytes)] [30, 33, 34]. Importantly, Brn-3b target genes control many essential cellular processes including proliferation, metabolism, differentiation and apoptosis, depending on the cell type and growth conditions [24, 26, 30, 35]. For instance, Brn-3b controls survival and differentiation in retinal ganglion cells by regulating sonic hedgehog, myostatin (Gdf8) and Pax4 [36, 37]. However, in epithelial-derived tumour cells, Brn-3b promotes cell proliferation by activating cell cycle proteins, cyclinD1 and CDK4 while repressing the Brca1 tumour suppressor gene [38, 39]. Yet in metabolic tissues including skeletal muscle and adipose tissues, Brn-3b is implicated in regulation of glucose homoeostasis by controlling expression of Glut4 and GSK3β, since reduction or loss of Brn-3b is linked to hyperglycaemia and insulin resistance [35].

Brn-3b is highly expressed in foetal hearts and while expressed at low levels in normal adult hearts, this TF is increased in adult hearts following acute injury (e.g. coronary artery ligation) [24] or in response to chronic stress e.g. angiotensin II treatment, which induces ventricular hypertrophy [26]. Recent studies using male Brn-3b KO mutant mice have shown that loss of Brn-3b caused subtle differences in contractility and cardiovascular function at baseline e.g. reduced arterial elastance and end systolic pressure volume relationship [26]. Moreover, Brn-3b appears to be necessary for normal adaptive responses in male hearts because angiotensin II treatment in Brn-3bKO mutants caused extensive fibrosis in the ventricular wall and remodelling around the coronary vasculature, which was linked to adverse functional responses including reduced cardiac output and ejection fraction [26].

Remodelling around Brn-3b KO coronary arteries pointed to potential roles for this TF in controlling vascular integrity and function^(*35*)^ especially since published genome wide association studies (GWAS) data have identified SNPs associated with coronary heart disease (CHD), coronary artery diseases (CAD) and cerebrovascular accident (CVA) within the genomic region chromosome 4q (31.2), containing the Brn-3b genomic locus [40–45]. However, to our knowledge, the expression and effects of Brn-3b in the vasculature are still unknown.

In this study, we confirmed Brn-3b expression in the aorta, with localisation to VSMCs. Studies using Brn-3b KO mice showed that loss of this TF caused significant histological and structural changes including small but consistent narrowing of the aorta, thickening of the tunica adventitia (TA) and disrupted Eln fibres in aortas from male mice. This was associated with increased wall stiffness and attenuated contractile responses to KCl, PE and U46619. RNA sequencing analysis and subsequent validation studies showed that loss of Brn-3b affected genes such as Cacna1d; S/ER Ca^2+^ channel, rayanodine receptor RyR2, Pln and reduced Atp2a1, which are implicated in controlling Ca^2+^ signalling and primary VSMCs cultured from Brn-3b KO aortas displayed abnormal Ca^2+^ responses to ATP. Similarly, reduced chaperone genes e.g. Hsph1, Hspb1, DnajA1 in Brn-3b KO aortas, which is linked to increased ER stress [20, 46] was associated with phenotypic switching and increased proliferation in Brn-3b KO VSMC cultures. Taken together, these results suggest that Brn-3b TF controls genes that are required for maintaining calcium homoeostasis, contractility and stress responses in VSMCs and loss of Brn-3b will lead to vascular dysfunction.

## Materials and methods

### Materials

General laboratory reagents: Merck (Nottingham, UK) and Sigma (Dorset, UK), unless otherwise stated. Primary antibodies: Brn3b-rabbit pAb (ab128849- now discontinued). Abcam-Cambridge, UK); Brn3b-rabbit pAb Biorbyt ORB576708; orb 1313 (now discontinued); GAPDH and β-tubulin (Cell Signalling Technology, USA) α-SMA (Ab7817;Abcam-Cambridge, UK); Col1a1 (ab88147Abcam, Cambridge, UK); Ki67- M7249 (Dako, Cambridgeshire, UK). HRP-conjugated secondary Ab, Dako (Cambridgeshire, UK).

#### qRT-PCR Primer Sequences

Brn-3b F - 5′ GAGAGAGCGCTCACAATTCC 3′;

Brn3b R- 5′ ATGGTGGTGGTGGCTCTTAC 3′

36b4 F- 5′ AGATGCAGCAGATCCGCAT 3′;

36b4 R- 5′ GTTCTTGCCCATCAGCACC 3′

Gapdh F- 5′ CTTCATTGACCTCAACTAC 3′;

Gapdh R 5′ AGTGATGGCATGGACTGTG 3′

Ryr1 F- 5′ GCAGGAGACGTACAGTCAGG 3′;

Ryr1 R – 5′ CAAGGATGTCTGCACGGAGT 3′

Ryr2 F-5′CCTACAGTGGTATGTATCTTTGCTGTCT3′;

Ryr2 R- 5′CTCTTGAAGGCCAACATC GAA 3′

Cacna1d- F-5′CGTGCCTTCCGAGTGTTAAG3′;

Cacna1d- R-5′GGACCATGGCTTTTATAATGGA 3′

Cacnb2 F- 5′TGGAGTCGACTTTTTGCCGA 3′

Cacnb2 R- 5′ TCCATAGGACTGTGCTCCGA 3′

Pln F- 5′ ACCGAAGCCAAGGTCTCCTA 3′;

Pln R- 5′ TAGCCGAGCGAGTGAGGTAT 3′

DnaJa1 F- 5′ CCGCTCACCGGCTGTAAA 3′;

DnaJa1 R- 5′ GGGTGGTACTTCAAGGCCAA 3′

Hspa8 F- 5′ CTGCTGCTATTGCTTACGGC 3′;

Hspa8 R- 5′ TCAAAAGTGCCACCTCCCAA 3′

Hsph1 F- 5′AACCCCAGATGCTGACAAAG 3′;

Hsph1 R- 5′GCAGCTCAACATTTACCACCT 3′

Atp2A1 F- 5′AAGGCTCGGGACATCGTT 3′;

Atp2A1 R- 5′GGATGTCTGCAGGGACTTTG 3′

ELNF1 F- 5′ GCTGATCCTCTTGCTCAACC

ELNF1 R- 5′ CAATACCAGCCCCTGGATAA

MMP-2 F- 5′TGCCCCCATGAAGCCTTGT3′

MMP-2 F- 5′TACAGCTGTTGTAGGAGGTGCC 3′

## Methods

### Experimental models

All animal experiments were carried out in compliance with UK Home Office regulations (Animals Scientific Procedures Act 1986) and approved by local UCL Ethics Review Board. Early studies used Wistar rats or wild-type (WT) C57Bl/6 mouse models purchased from commercial companies (Harlan UK). Later studies with knockout (KO) mice and WT littermate controls were obtained by crossing Brn-3b heterozygotes C57Bl/6 strains. For all experiments involving animal models, a priori sample power calculations were carried out by using multiple endpoints with online tools e.g. IACUC power calculator, to determine the sample sizes required to achieve statistical significant (e.g. 80% power and 95% confidence limits) to detect prespecified effects. Randomisation studies and blinding during analysis were not possible for these experiments because of the requirement for consistency in processing diverse data sets with different endpoints.

### Histological staining

To analyse for morphological and structural changes in the aortic sections, histological staining was undertaken using Haematoxylin and Eosin (H&E) staining; Masson′s trichrome staining (Abcam-Cambridge, UK) or oil red O staining (Sigma, UK). For these studies, formalin-fixed, paraffin-embedded aortic sections from age matched Brn-3b KO and WT mice, were dewaxed, rehydrated and stained according to the manufacturer’s protocol. To quantify TA thickness matched aortic sections were analysed using NDPview-2 software (Hamamatsu, Japan), by undertaking ~50 equidistant measurements of the aortic wall. Data represents mean ± SD from ≥6 matched, independent WT or Brn-3b KO aortic sections.

#### Protein preparation and quantification

Total protein extracts were prepared either from mouse aortas or primary VSMC cultures as follows. For aortic samples, intact aortas dissected from 2 to 3 mice in each group (WT controls or Brn-3b KO mutants) were processed using standard protocols [26] i.e. dissociated snap frozen tissues resuspended and processed in RIPA buffer. Total protein content was quantified using BCA (bicinchoninic acid) kit (Merck, UK) and ~30 μg/well protein extracts were resolved on SDS-PAGE gels then transferred onto PVDF membranes overnight [24]. Following incubation (1 h at RT) with block buffer [4%milk + phosphate buffered saline + 0.1% Tween-20 (PBST)], and primary antibody for 2 h at RT or overnight at 4 °C (optimised for specific antibodies); membranes were washed (X5) then incubated with HRP-conjugated 2nd antibody (1 h; RT). Signals were developed using enhanced chemiluminescence (BioRad, UK) on Syngene G:BOX imager and protein quantification done out using FiJi (ImageJ) software. Variation in protein loading was adjusted with β-tubulin (housekeeping protein).

#### Co-immunostaining using colorimetric or fluorescent detection

Immunostaining for protein localisation was done using paraffin-embedded tissue sections or cultured cells. Tissue sections were dewaxed and rehydrated prior to antigen retrieval (0.01 M sodium citrate pH6.0, 10 min), and single or double immunostaining protocols undertaken using Vectastain Elite ABC Kit (Vectorlabs) or ImmPRESS® Duet Double-Staining Kit, (according to the manufacturer’s protocol [25, 26]). Primary antibodies were incubated in a humidified chamber, either 2 h (RT) or overnight (4 °C) and control samples were incubated with second antibody only. The manufacturers protocol was modified to include additional washes (5 PBST; 5 min) after which detection was undertaken using Vectastain Elite ABC Kit (Vectorlabs) or ImmPACT® DAB EqV Substrate (HRP, brown); ImmPACT Vector® Red Substrate (AP, magenta). Following graded ethanol dehydration, cover-slipped slides were imaged using the Hamamatsu Nanozoomer (Hamamatsu, Japan) then analysed using NDP view 2 software.

Fluorescent immunostaining of cultured cells was undertaken as previously described [24]. Briefly cells were fixed in 4% PFA and permeable if necessary. Following incubation with block solution (20% goat serum in PBST) antibodies were incubated either overnight at 4 °C or 2 h at room temperature. Following 5× washes in PBST and incubation with appropriate fluorescent tagged, secondary Ab, cells were imaged using fluorescent microscopy and Leica software for analysis (Leica DMi8, LAS X).

#### Transmission electron microscopy

Transmission electron microscopy (TEM) were undertaken using resin embedded sections prepared from Brn-3b KO mutant aortas and wild-type controls using well established methods. Briefly, aortas were perfused and fixed in 100 mM cacodylate buffer containing 2.5% glutaraldehyde overnight at 4 °C. Samples were transferred to 1% osmium tetroxide, dehydrated in an ethanol series, and then embedded in araldite resin (Electron Microscopy Sciences, Hatsfield, PA). 0.5 μm sections were stained with toluidine-blue and analyses using light microscopy. For electron microscopy, ultra-thin sections (70–80 nm) were cut and set on 200-mesh copper. The grids were counterstained with lead citrate for 3 min and imaged on a Jeol 1400 electron microscope with a Rio Gatan camera.

#### RNA sequencing and data analysis

For RNA sequencing, total RNA was prepared from aortas taken from Brn-3b KO male mutants and age-matched wild-type controls (>3/set). Quality controls (QC) was done using NanoDrop and Agilent Bioanalyser 2100 before library preparation was carried out using KAPA mRNA HyperPrep Kit (p/n KK8580) according to manufacturer’s instructions. During this process, highly purified mRNA, fragmented by chemical hydrolysis and primed with random hexamers, was used for RNA-dependent cDNA synthesis with “A-tailed” at 3′ end, to prevent self-ligation and adaptor dimerisation. Amplification and first strand library preparation was undertaken using adaptors for PCR with high fidelity polymerase and libraries were sequenced on the NextSeq 500 instrument (Illumina, San Diego, US) using either a 43 bp or 81 bp paired end run with general yield of ~15 million reads per sample. De-multiplexed data converted to Fastq files (Illumina’s bcl2fastq Conversion Software v2.19) were used for alignment with the reference genome using STAR (v2.5b) and aligned data were deduplicated (Picard Tools v2.7.1) and reads per transcript counted by FeatureCounts (v1.4.6p5). Ensembl ID for each gene was determined using g:Profiler and this data was then used for normalisation, modelling and differential expression analysis using the iDEP.93 software platform, which connects multiple R/Bioconductor packages with annotation and pathway databases to provide comprehensive analysis of RNA sequencing data

#### Data analysis using iDEP.93 platform

Preprocessing was done to filter genes with ≥0.5 counts per million in at least one sample inclusion in the analysis using EdgeR (default settings CPM-0.5, pseudo-count c-4) and principal component analysis was undertaken using PC1 and PC 3 to visualise the relationship between sample groups based on sample variance. For K-means clustering analysis, read counts were transformed to log_2_ and the 2500 most variable genes (based on standard deviation ranking) were separated into one of four clusters, based on functionality, molecular and biological processes with students t-test used to calculate the values for enriched pathways within each cluster. Kyoto Encyclopedia of Genes and Genomes (KEGG) pathway analysis provided a systematic analysis of pathways associated with genes in each cluster (adjusted *p* value). Similarly, Gene Ontology (GO) database platforms provided information on the association of genes within each cluster with specific biological processes, molecular function and cellular components. The Disease.Jensen.DISEASES database also linked to this platform, was used to identify diseases associated with specific subsets of genes, depending on adjusted *p* values. Gene Set Enrichment Analysis (GSEA) was undertaken to identify genes that were either up- or down-regulated in Brn-3b KO vs WT, using the DESeq2 method, which calculated the *p* values using the Wald test and used the read count data (unlike K-Means Clustering which uses transformed data). Analyses were undertaken using absolute values of fold changes in the pre-ranked mode (preranked fgsea) and were based on comparison between genotypes i.e. KO vs WT, with 15–2000 gene set (min; max), pathway significance cut-off of 0.1 false discovery rate (FDR) and minimum fold change of 1.5, with top 30 pathways selected. Normalised enrichment score (NES) accounted for gene set sizes and correlations between gene set and expression data, with positive and negative enrichment scores (ES) indicating correlation with Brn-3b KO and WT phenotypes respectively. KEGG and GO pathway analyses were used to determine functional changes associated with up- or down-regulated gene sets.

Differential Expression of Genes (DEG) was also carried out using DESeq2 method to identify genes with significant and concordant differences in a-priori gene sets in the different variables (Brn-3b KO vs WT). The minimum fold change was set at 1.5 and with 0.10 FDR cut off. WT values were used as the control baseline for the analyses. Functionality of up and/or downregulated was determined using g:Profiler.

#### Primary VSMC cultures

Primary VSMC cultures were prepared from freshly dissected Brn-3b KO and WT aortas as described [47]. Briefly, excess perivascular tissue and tunica adventitia were carefully removed from isolated aortas (to prevent contamination with fibroblast of perivascular adipose tissue (PVAT)) and ~2 mm of cleaned aortic rings were carefully placed into a T25 flask, in large drops of growth medium (DMEM-F12 + 1% Penicillin-Streptomycin +15%foetal calf serum) and left to attach to the flask. After 5–10 min, culture medium was gently added to cover flask, which was then placed in a humidified incubator (37 °C and 5% CO_2)_. Media were changed every 2–3 days until cells were confluent and ready for experiments (up to 10–14 days).

### Gene validation: RNA extraction, cDNA synthesis and quantitative polymerase chain reaction (qRT PCR)

RNA was extracted from snap-frozen aortas taken from Brn-3b KO and WT aortas. Tissues were crushed in liquid nitrogen and homogenised in RLT buffer then RNA was extracted using Qiagen RNeasy mini kit (Qiagen, Manchester, UK) or TRIZOL® Reagent (Invitrogen). Since Brn-3b(s) is only encoded by exon 2, it was not possible to use primers spanning the intron. As such, it was necessary to remove all contaminating genomic DNA prior to cDNA synthesis, which was achieved using RNAse-free DNAse1 (Promega, Southampton, UK). 1 μg of total RNA from each sample was used for cDNA synthesis, using Superscript™ II Reverse Transcriptase (Invitrogen, UK), according to the manufacturer’s protocol.

Validation studies to confirm changes in RNA sequencing data for selected genes was undertaken using q-RT-PCR with Eppendorf Mastercycler using SYBR Green chemistry. Primers designed for each target gene were used to amplify cDNA for specify genes using independent WT or KO aortic RNA. Housekeeping genes e.g. GAPDH and 36B4 were used correct for RNA variability between samples and a reference sample was included to facilitate use of ΔΔCT method to calculate fold changes in relation to controls samples. Statistical analysis was undertaken using results from multiple experiments using independent aortas with student’s *t* test used to show significance, *p ≤ 0.05.

### Wire myography for contractility studies in Brn-3b KO and WT aortic vascular rings

Thoracic aorta from Brn-3b KO and WT mice were prepared by removing perivascular vascular tissue. The cleaned aortae were used to generate vascular rings for wire myography during which tissues were bathed in Krebs solution at 37 °C with a 95% O_2_ and 5% CO_2_ environment. Vascular rings were mounted on the myograph (Danish Myo Technology, Hinnerup, Denmark) and DMT normalisation module used for isometric tension recordings. At baseline, the optimal pre-tension condition established by stretching to 95% internal circumference with resting transmural pressure of 100 mmHg (IC100). Following equilibration and normalisation of vascular reactivity, constricting cumulative dose-responses were measured following treatment with potassium chloride (KCl, 7.50–90 mM/L), phenylephrine (PE, 100 nM/L to 100 μM/L) (Sigma-Aldrich, Missouri, USA) and U-46619 (0.1–25 nM/L) (Cambridge Biosciences, Cambridge, UK). Results shown at raw force (mN) [48, 49].

#### Calcium imaging using VSMC

For these studies, primary VSMC cultures derived from single WT and Brn-3b KO mouse aortas were plated into glass FluoroDish petri dishes (WPI World Precision Instrument, Thermo Fisher Scientific, UK) and grown to ~70% confluency. Prior to signal imaging and analysis, cells were transferred to calcium free growth medium then loaded with fluo-4-AM dye (Thermofisher Scientific) by adding to HBSS in presence of 0.02% pluronic F-127 and incubating at 37 °C in 5% CO_2_ for 30 min, according to manufacture instructions in. Cells were washed twice in HBSS and maintained in 900 uL of HBSS at 37 °C in 5% CO_2._ Basal fluorescence signals were measured and recorded using the Zeiss LSM 880 Observer Z1, Airyscan confocal microscope after which, ATP was carefully added to the dish to a final concentration of 100 uM and changes in fluorescence intensity were recorded for 400 at 1 frame per sec. Since each sample was analysed individually, measurements were done by alternating WT and KO for pairwise comparison. Time-lapse movies were analysed using ImageJ with >10 cells analysed for each sample. For each cell, the changes in signals over time were expressed as fold change of the fluorescence at baseline and for each VSMC culture, a minimum of ten cells were analysed to obtain the average signals for that sample. The final data represents data from six independent studies carried out in individual aortas.

#### Analysis of VSMC proliferation rates using BrdU incorporation studies

The rate of proliferation was measured by determining DNA synthesis using 5-bromo-2- deoxyuridine (BrdU) Cell Proliferation ELISA Kit (Abcam, Cambridge, UK) and was carried out according to the manufacturer’s protocol. Briefly, sub confluent VSMC cultures from Brn-3b KO or WT aortas were pulse labelled with BrdU for 2 h. Cells were fixed for 30 min then washed prior to addition of the primary detector antibody for 1 h at room temperature (RT). Following washes, peroxidase conjugated second antibody was incubated (30 min RT). Following incubation with the peroxidase substrate (30 min, RT), the reaction was stopped and signals were quantified using spectrophotometer microtitre plate reader set at dual wavelength (450/550 nm).

#### Statistical analysis

Statistical analysis was carried out using GraphPad Prism or Excel. To determine statistical significance within different dataset analysis were undertaken using T-test, F-test or 2 way ANOVA with Bonferroni post-test. T-test was used to determine *p* value and standard deviation while F test were used to compare variances and determine significance of differences. Two-way ANOVA also provided detailed comparisons between groups with additional data including sum-of-squares main square and analysis of sources of variation.

## Results

### Brn-3b expression and localisation in the aorta and primary VSMC cultures

**Fig. 1:**
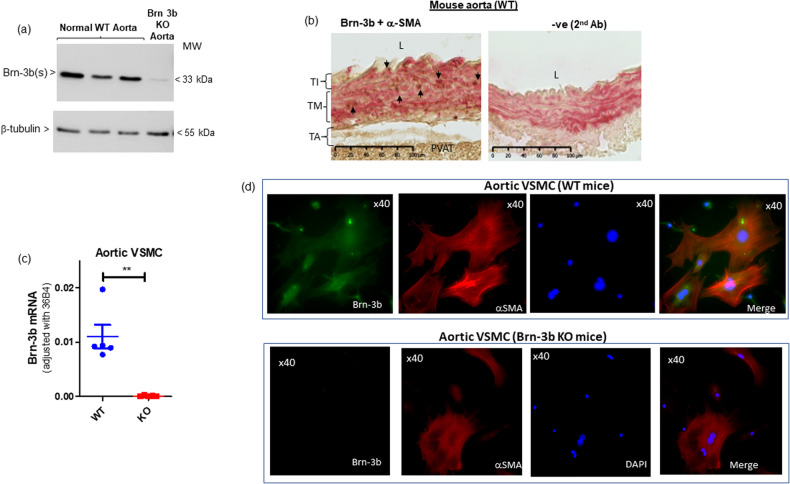
Brn-3b expression in aortic protein extracts and VSMC cultures. **a** WB showing expression of the shorter Brn-3b(l) protein in aortic extracts prepared from pooled WT aortas (dissected from 2–3 mice) compared with pooled aortas from Brn-3b KO mutants (negative control). β−tubulin blots indicate variation in protein loading. **b** Representative images of immunostained WT aortic sections showing co-localisation of Brn-3b protein (brown stain) with VSMC marker, αSMA (magenta). Negative control = 2nd Ab and α-SMA. Different layers of the aorta: TI tunica intima, TM tunica media, TA tunica adventitia. **c** quantification of Brn-3b mRNA in primary VSMC cultures prepared using aortas from WT mutant n-3b KO mice (*n* = 5 independent cultures). Analyses were carried out using student’s t-test. **d** Representative immunofluorescent images of aortic VSMCs from WT mice (top panel) or Brn-3b KO mice, co-stained with Brn-3b (green) and α−SMA (red). DAPI mount indicates cell nuclei (blue).

To investigate expression and roles for Brn-3b in BV, pooled aortas from wild-type (WT) mice were used to analyse for protein expression using western blot (WB) analysis. Fig. 1a shows that Brn-3b(s) protein isoform was readily detectable in pooled aortic protein extracts from WT mice but not in Brn-3b KO aortic extracts while β-tubulin blot is included to show variability in total cellular protein. It should be noted that only the shorter Brn-3b(s) isoform was detected in aortic tissue extracts (full blot in Supplementary Fig. 1 also shows positive control, testis, primarily expressing the Brn-3b(l) isoform).

Brn-3b protein localisation in aortic sections was also analysed by undertaking co-immunostaining studies with α-SMA antibody used to mark VSMCs. Studies were carried out using aortic sections from WT and Brn-3b KO aortas. As shown in Fig. 1b, Brn-3b protein (brown stain) was detected mainly to the medial layer in WT aortic sections, where it was co-localised with the VSMC marker, α-SMA (magenta) but not in control WT sections stained with secondary Ab only.

To confirm Brn-3b expression in VSMCs, primary cultures were isolated and grown from WT or Brn-3b KO mouse aortas and either used for quantification (qRT-PCR) or localisation studies (co-immunostaining). qRT-PCR data shows that Brn-3b mRNA was detectable in VSMC cultures prepared from WT but not Brn-3b KO aortas (Fig. 1c). Similarly, co-immunostaining studies confirmed Brn-3b antibody staining (green) colocalized with α-SMA (red) in VSMC derived from WT aortas (top panel). Comparison with DAPI staining (blue) showing cell nuclei indicated that Brn-3b was expressed in the nuclear and perinuclear regions in aortic VSMCs. As expected, Brn-3b was undetectable in VSMC from Brn-3b KO mutant aortas (bottom panel) in cells that express α-SMA (red).

### Abnormal structural and histological changes in Brn-3b KO aorta

**Fig. 2:**
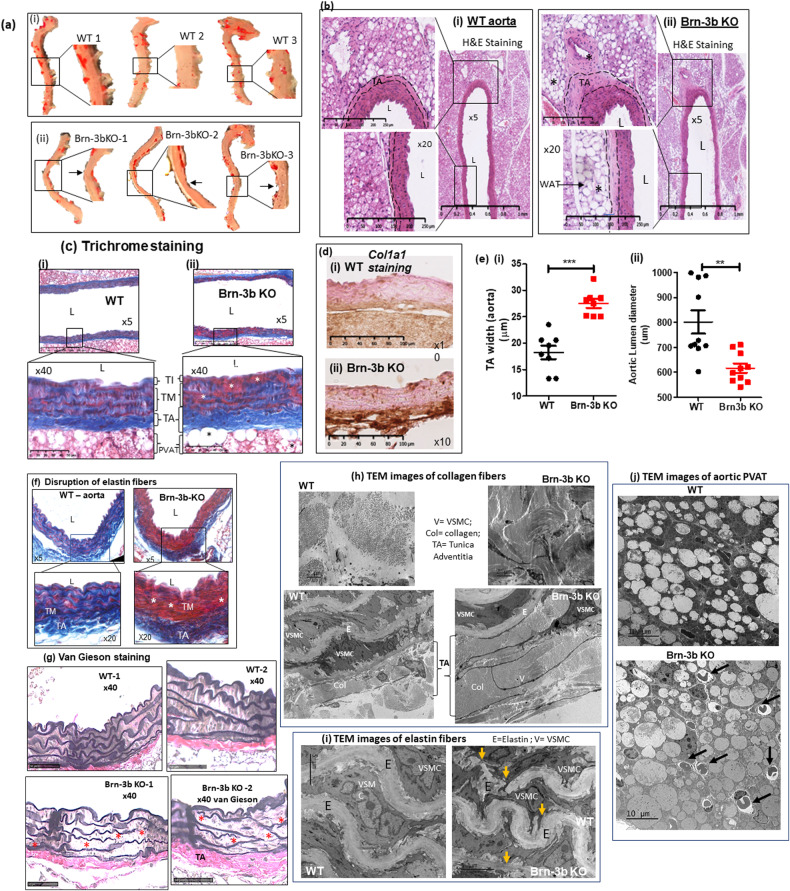
Histological changes in Brn-3b KO aorta compared with WT. **a** Representative images of intact aortas taken from WT or Brn-3b KO mice, after staining for oil red. Images shown at X5 magnification with boxed areas expanded to highlight narrowing/constriction in Brn-3b KO aortas and corresponding WT aortas. **b** H&E staining of longitudinal aortic sections from WT (i) or Brn-3b KO mice (ii). 20× magnification of selected areas shows increased adventitial thickness highlighted by dotted lines (arrows indicate specific areas of thickening in KO aortas; * highlights increased WAT deposition) in Brn-3b KO but not WT aortic sections. L lumen. Images (**b**–**g**) were captured using Hammamatsu Nanozoomer imaging system and shown at ×5; ×20 and ×40 magnification. TI tunica intima, TM tunica media, TA tunica adventitia, WAT white adipose tissue, PVAT perivascular adipose tissue. **c** Representative images showing Masson’s trichrome staining of longitudinal aortic sections from either (i) WT or (ii) Brn-3b KO mice which highlights the increases in thickness of tunica adventitia (TA) layer and elastin disruption in tunica media (TM) of Brn-3b KO aortas (*), when compared with WT controls. Red staining represents cytoplasm; dark purple/black shows cell nuclei; blue staining indicates ECM protein (e.g. collagen) deposits. **d** Representative images showing immunostaining for collagen protein, Col1a1, in WT and Brn-3b KO aorta sections using DAB immunostaining protocol, with intense brown staining indicating increased expression in aortic sections from Brn-3b KO mice compared with WT controls. **e** (i) Quantification of TA width thickness in representative aortic sections, with each point representing the mean values from eight independent mice within each group. Data represents mean and standard deviation (±SD) with significance (***p < 0.001), determined by students *t* test. (ii) Graph showing differences in aortic lumen diameter from WT or Brn-3b mice (*n* = 8 independent aortas/group). Analysis done using students t-test. **f** Masson’s trichrome stained aortic section highlighting disruption of elastin fibres in Brn-3b KO mutants; indicated by fragmented blue staining (*), compared with WT aortas. **g** van Gieson staining of elastin fibres (dark brown) in aortic section from WT or Brn-3b KO mice showing disruption of elastin fibres in Brn-3b KO aortas (arrowheads) when compared with WT aortas. Magnification is shown at 5× (top panels) and 40× magnification (bottom). **h**–**j** Transmission electron microscopy images showing differences between aortas taken from WT and Brn-3b KO mice. **h** Increased collagen fibres in the Tunica adventitial (TA) layer with Brn-3b KO aortas showing significantly thicker TA with more compact arrangement of collagen fibres when compared with WT controls. **i** Representative images of the tunica media (TA) showing elastin fibres and VSMCs found between the elastic fibres (E) are indicated. Disruption of elastin fibres in Brn-3b KO aortas are indicated by yellow arrows, when compared with WT aortas. **j** Images showing aortic perivascular adipose tissue (PVAT) surrounding the aorta taken from either WT or Brn-3b KO mice. Black arrows indicate larger numbers of infiltrating lymphocytes in Brn-3b KO PVAT when compared with WT controls.

Since loss of Brn-3b was linked to extensive remodelling around the coronary arteries in male Brn-3b KO hearts [26], we next analysed for structural changes in aortas from male Brn-3b KO mice, when compared with WT controls. Fig. 2a shows that aortas from Brn-3b KO mice displayed small but consistent narrowing/coarctation in the descending aortas, when compared with age and gender-matched WT controls. H&E staining of aortic sections also showed marked histological changes in Brn-3b KO aortas (Fig. 2b), which included thickening of the adventitial (TA) layer and increased white adipose tissue (WAT) deposits in PVAT, when compared with WT aortas. Such changes were also confirmed using Masson’s trichrome staining, with Fig. 2 showing increased deposition of ECM proteins (blue) in the thickened adventitial layer (TA) of Brn-3b KO aortas (ii), when compared with WT aortas (i). These differences were also confirmed by quantifying changes in TA thickness and lumen diameter in transverse aortic cross-sections from independent age-and gender matched WT or Brn-3b KO aortas (*n* > 6). Figure 2d(i) shows that Brn-3b KO aortas displayed significant increases in TA thickness but reduction lumen diameter, when compared with WT aortas [Fig. 2d(ii)]. Immunostaining of aortic sections using collagen 1A1 (Col1a1) antibody also confirmed increased Col1a1 staining in the TA layer of Brn-3b KO aortas, when compared with WT controls (Fig. 2e).

Interestingly, analysis of trichrome staining aortic sections at high magnification also showed significant changes in elastin fibres in Brn-3b KO aortas when compared with WT (Fig. 2f). Therefore, van Gieson staining was carried out on aortic sections to analyse for changes in elastin fibres. Fig. 2g confirmed that Brn-3b KO aortas displayed disrupted elastin fibres compared with WT controls.

Such significant morphological and structural changes in Brn-3b KO aortas were also confirmed at the ultrastructural level, using images from TEM (Fig. 2h, i). Fig. 2h shows increased density and compaction of collagen fibres in the thicker TA layer of Brn-3b KO aortas compared with WT aorta. Similarly, disruption in the elastin lamina in mutant aorta can be clearly seen in Fig. 2i while Fig. 2j shows differences in fat cells but also highlights infiltration of immune cells (arrows) in aortic PVAT from Brn-3b KO aortic sections that was not evident in PVAT from WT aortas. These results confirmed that structural and histological changes linked to morphological abnormalities in Brn-3b KO aortas were associated with ultrastructural defects that could profoundly alter aortic structure and function.

### Contractile changes in Brn-3b KO aorta

**Fig. 3:**
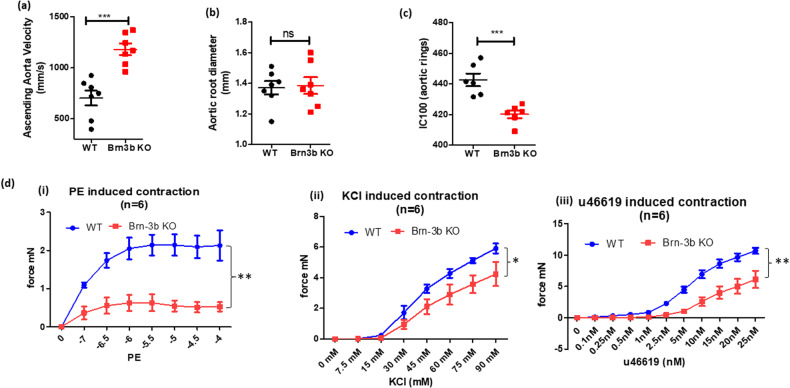
Analysing contractile responses in vascular rings from Brn-3b KO aortas. Echocardiography data showing changes in ascending aortic velocity (**a**) and measurement of aortic root diameter in male Brn-3b KO mice (**b**), when compared with age-matched WT controls. **c** Graph showing IC100 measurement (from wire myography) to measure differences in elastic properties of vascular rings from WT and Brn-3b KO mice at baseline. **d** Summary of data showing responses of thoracic aortic vascular rings from Brn-3b KO aortas and WT controls, following treatment with cumulative concentration of (i) KCl, (ii) PE and (iii) PGH2 prostaglandin analogue, u46619. Graphs show changes in force of contraction (DmN) elicited following different treatments, as shown. Data represents mean and standard error of measurements from vascular rings taken from 6 independent Brn-3b KO and WT aortas. Statistical significance was determined using two-way ANOVA analysis.

To determine how structural changes may affect aortic blood flow in Brn-3b KO aortas, echocardiography was undertaken using Visualsonics Vivo 2100 to analyse ascending aortic velocity and aortic root diameter in male Brn-3b KO mutants and age-matched WT controls. Figure 3a shows that ascending aortic velocity was significantly increased in Brn-3b KO mutants, when compared with WT controls, despite no significant differences in aortic root diameter (Fig. 3b), which suggested that loss of Brn-3b may contribute to changes in aortic contractility.

Therefore, wire myography was next used to analyse for differences in elastic properties and contractile responses in aortic rings from Brn-3b KO mutants and WT controls using. For these studies, baseline wall stiffness in aortic rings was established using the normalisation method whereby vascular rings were pre-stretched to 95% of internal circumference with resting transmural pressure of 100 mmHg (IC 100). As shown in Fig. 3c, the IC100 values were significantly lower in Brn-3b KO vascular rings which may be linked to changes in lumen diameter (Fig. 2d(ii) but could also reflect differences in the elastic properties of mutant blood vessels.

We next analysed for changes in contractile responses of pre-stretched, equilibrated vascular rings from Brn-3b KO and WT aortas by adding cumulative dose of known mediators of vascular contraction including phenylephrine (PE), prostaglandin analogue, U46619, or potassium chloride (KCl) [50–52]. Pooled data from independent aortas, taken from six WT and six Brn-3b KO mice, (Fig. 3d) showed that, as expected, vascular rings from WT aortas displayed dose-responsive increases in force of contraction following treatment (i), with PE causing the most significant responses. In contrast, contractile responses in Brn-3b KO aortas were considerably blunted following PE treatment, indicating that mutant aortas were unable to contract significantly. Similarly, treatment with U46619 (ii), and KCl (iii) also induced strong contraction of vascular rings from WT while responses in Brn-3b KO aortas remained significantly attenuated. These highly reproducible results from independent aortas suggests that pre-constriction of mutant aortas upon loss of Brn-3b may prevent subsequent contractile responses to PE, U46619 or KCl.

### RNA sequencing analysis to identify genes altered in Brn-3b KO aortas

**Fig. 4:**
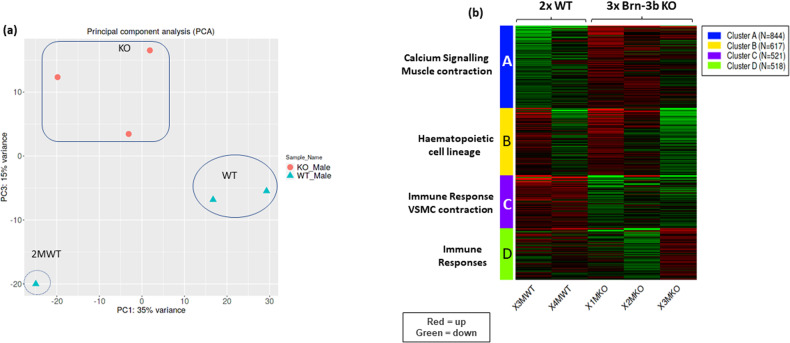
Analysis of RNA seq data using iDEP9.1 software. **a** Principal Component Analysis (PCA) of all data obtained from RNA sequencing of WT and Brn-3b KO aortas. Samples from all three KO aortas (orange dots) were clustered together (boxed area) but only two WT samples (oval) showed similar patters while the WT sample 2 M (circled), was identified as an outlier with significant differences in both PC1 and PC3 components and was excluded from analyses. **b** Heat map showing the four clusters arising from K-means clustering of the top 2500 from two WT and three KO samples (excluding outlier sample, 2 M WT). KEGG pathways associated with enriched genes within each cluster are shown i.e. Red = upregulated genes; green = down regulated genes.

To understand the molecular basis for such changes in Brn-3b KO aortas, RNA sequencing analysis was undertaken to identify genes that were differentially regulated upon loss of Brn-3b in the aorta. Following in-depth analysis of data using iDEP.91 software [53], principal component analysis identified one WT outlier (Fig. 4a) but K-means analysis of the top 2500 differentially regulated genes showed that the same gene clusters were identified when using all datasets (Supplementary S-Fig. 1) or when the outlier was excluded (Fig. 4b). However, the outlier caused some skewing in subsequent analyses to identify up- and down regulated genes so was omitted for later analyses with selected genes validated in multiple, independent mRNA from WT or Brn-3b KO aortas.

The most consistent and significant gene expression changes between WT and mutant aortas were observed in 2 clusters, A and C (Fig. 4 and Supplementary Fig. 1b; Supplementary Table 1b). KEGG pathway analysis showed that genes in cluster A (844 genes; adjusted *p* values < 8.0e-0.3) were strongly associated with calcium signalling (adjP 3.4e-05) and muscle contraction (adjP 3.4e-05) (Fig. 4) that directly affect vascular function while GO analysis confirmed that genes within this cluster were mainly associated with the plasma membrane or ion channels in the S/ER and participated in biological processes including sarcomeric structures and ion transport, (Supplementary Table 1c, d). Since Ca^2+^ signalling genes are also implicated in controlling cardiac contractility and function [54–56], it is unsurprising that cardiac pathways including adrenergic signalling, cAMP signalling were also identified (s-Table 1e). Interestingly, other affected genes within this cluster were linked to regulation of circadian entrainment (adjP 1.8e-03) and metabolic processes (adjP 5.3e-03), which can indirectly contribute to vascular dysfunction [57–59] (Supplementary Table 1). In contrast, genes in cluster C (521 genes; adjusted *p* values < 2.6 e-0.3) were mainly linked to regulation of VSMC contraction (adjP 3.6e-03) but also immune responses (adjP 2.6e-03), which can indirectly alter vascular function (Supplementary Table 1a) [12, 60, 61]. GO analysis also confirmed that cluster C genes were implicated in VSMC contraction and muscle development/differentiation. Affected genes in cluster B (617 genes; adj *p* values < 7.5e-0.3) and D (542 genes; adj *p* values < 5.3e-0.3), were linked to haematopoietic cell lineage and immune responses respectively but the sample-to-sample variation limited any meaningful interpretation of this data.

### Identification of up- and down-regulated genes

GSEA was next combined with KEGG pathway and GO analyses to identify how genes that were specifically up- or down-regulated in Brn-3b KO aortas affected key functional pathways that were linked to essential biological processes. Table 1a shows the largest number of upregulated genes in Brn-3b KO aortas (158 genes) were implicated in Ca^2+^ signalling and related pathways (cAMP signalling and β-adrenergic signalling) with significant normalisation enrichment scores (NES+ 1.51) while 93 genes (NES + 1.45) were implicated in VSMC contraction. Supplementary Table 1 (s-1) shows the list of genes within each cluster. In support of essential findings for such differential gene expression, GO analyses of biological function and cellular component showed that genes within these groups encoded for ion channels (S/ER or plasma membrane) and regulatory proteins that were involved in biological and molecular processes such as Ca^2+^ signalling and muscle contractility (s-Table 2). Interestingly, genes affected by loss of Brn-3b were also implicated in human diseases since Jensen.disease pathway analysis (Table 1c) showed that the most significant pathways affected by genes upregulated in Brn-3b KO aortas were hypertension (169 genes; NES + 1.48) CAD (128 genes; NES + 1.53).

**Table 1:** Summary of Gene Set Enrichment Analysis (GSEA) used to identify pathways affected by loss of Brn-3b in the aorta.

a			
GSEA analysis: Upregulated genes (KEGG Pathways)	NES	Genes	adj.Pval
Calcium Signalling and Muscle contraction			
**Calcium signalling pathway**	**1.5097**	**158**	**3.30E-04**
CAMP signalling pathway	1.4551	146	3.30E-04
Adrenergic signalling in cardiomyocytes	1.5349	119	3.30E-04
Oxytocin signalling pathway	1.4507	114	8.00E-04
**Vascular smooth muscle contraction**	**1.4459**	**93**	**4.20E-03**
Neuroactive ligand-receptor interaction	1.4483	90	4.20E-03
Dilated cardiomyopathy	1.6415	79	3.30E-04
Hypertrophic cardiomyopathy	1.6252	74	3.30E-04
Aldosterone synthesis and secretion	1.4714	72	5.10E-03
Cardiac muscle contraction	1.7434	65	3.30E-04
Arrhythmogenic right ventricular cardiomyopathy	1.6189	64	3.30E-04
**Circadian pathways**			
Circadian entrainment	1.6437	68	3.30E-04
Circadian rhythm	1.8517	25	3.30E-04
Immune response and Haematopoiesis			
Hematopoietic cell lineage	1.4943	57	6.20E-03
IL-17 signalling pathway	1.4874	56	7.70E-03
Viral protein interaction - cytokine & cytokine receptor	1.5198	43	9.40E-03
Malaria	1.4702	29	6.20E-02
**Metabolic Processes**			
Pancreatic secretion	1.4943	61	5.50E-03
Cortisol synthesis and secretion	1.4363	47	3.60E-02
Regulation of lipolysis in adipocytes	1.4939	46	1.40E-02
GnRH secretion	1.528	44	8.50E-03
Long-term depression	1.4447	44	3.70E-02
Metabolism of xenobiotics by cytochrome P450	1.6406	39	1.80E-03
Chemical carcinogenesis	1.656	38	1.50E-03
Drug metabolism	1.5963	36	5.10E-03
Type II diabetes mellitus	1.4488	34	6.20E-02
Tryptophan metabolism	1.6074	25	1.40E-02
Retinol metabolism	1.6941	22	5.10E-03
One carbon pool by folate	1.4936	15	1.30E-01

b			
GSEA analysis Down regulated (KEGG Pathway)	NES	Genes	adj.Pval
**VSMC and muscle contraction**			
Regulation of actin cytoskeleton	−1.7531	40	2.0e-01
Vascular smooth muscle contraction	−1.6927	35	2.3e-01
Apelin signalling pathway	−1.7974	33	2.0e-01
**Immune response and Haematopoiesis**			
Chemokine signalling pathway	−1.9784	37	8.4e-02
Cytokine-cytokine receptor interaction	−1.7391	37	2.0e-01
IL-17 signalling pathway	−2.0966	16	8.4e-02
Alzheimer disease	−2.0803	15	8.4e-02
Hematopoietic cell lineage	−1.8507	15	2.0e-01
Human cytomegalovirus infection	−1.6782	41	2.3e-01
**Other processes**			
Oxytocin signalling pathway	−1.6876	34	2.3e-01
Prostate cancer	−1.9581	17	1.7e-01
Bladder cancer	−1.8539	10	2.0e-01

(c): JENSEN Disease pathways: Upregulated genes			
GSEA analysis: KO vs WT	NES	Genes	adj.Pval
Hypertension	1.4752	169	4.20E-04
Coronary artery disease	1.5283	128	4.20E-04
Arthritis	1.5689	115	4.20E-04
Cardiomyopathy	1.67	75	4.20E-04
Obesity	1.4581	68	1.50E-02
Congenital heart disease	1.4512	50	5.10E-02
Pneumonia	1.5689	37	1.50E-02
Major depressive disorder	1.4563	35	8.10E-02
Hyperinsulinism	1.4617	33	8.50E-02
Dilated cardiomyopathy	1.6009	31	1.50E-02
Distal arthrogryposis	1.7376	30	7.30E-04
Atrial fibrillation	1.5199	30	5.50E-02
Infertility	1.4683	29	9.40E-02
Eosinophilia	1.4631	27	1.00E-01
Pulmonary embolism	1.4986	23	9.40E-02
Meningitis	1.5829	22	5.20E-02
Hemochromatosis	1.5156	21	9.40E-02
Lymphedema	1.5966	20	5.20E-02
DOID:9917	1.5509	20	8.00E-02
Polyneuropathy	1.513	20	9.40E-02
Hyperhomocysteinemia	1.4927	20	1.10E-01
Leukopenia	1.4853	19	1.20E-01
Malignant hyperthermia	1.9492	18	4.20E-04
Pre-eclampsia	1.5755	17	8.00E-02
Candidiasis	1.4921	17	1.20E-01
Hypertrophic cardiomyopathy	1.6314	16	5.10E-02
Essential tremor	1.4935	16	1.20E-01
Hyperthyroidism	1.654	15	4.60E-02
Primary cutaneous amyloidosis	1.5242	15	1.10E-01
Hypokalemia	1.5129	15	1.20E-01

GSEA data showing functional KEGG pathways associated with genes that were either (a) upregulated or (b) down regulated in Brn-3b KO aortas, when compared with WT controls. Significance was determined using the normalisation enrichment scores (NES), number of genes and adjusted *p* value. NES [(+) = increased expression; (−) = down-regulated genes in Brn-3b KO tissue. (c) Summary of Jensen.disease pathway analysis to identify effects caused by genes that were significantly and when compared with WT controls differentially expressed genes in Brn-3b KO mutants.

Bold text represent the subheadings to highlight distinct pathways affected in mutant aorta compared with WTcontrols.

Consistent with K-means cluster analysis, genes upregulated by loss of Brn-3b were also implicated in pathways that can indirectly affect vascular function including circadian rhythm and entrainment, (68 genes; NES + 1.64); immune function (56 genes; NES + 1.48 that included IL-17 signalling, cytokine receptor interaction, haematopoietic cell lineage), and metabolic diseases (type II diabetes mellitus) (34 genes; NES + 1.45), (Table 1a and Supplementary Table 2a).

On the other hand, genes down-regulated in Brn-3b KO aortas were implicated in controlling VSMC function and related processes (Table 1b), with 68 down-regulated genes (NES −1.64) linked to VSMC contraction; 40 genes (NES-1.75) associated with regulation of actin cytoskeleton and 33 genes (NES −1.79) linked to Apelin signalling pathway. Other down-regulated genes were linked to immune responses [37 reduced genes (NES −1.98) in chemokine signalling and 16 genes (NES −2.09) linked to IL-17 signalling, indicating complex but, as yet unknown functions for Brn-3b in controlling vascular functions.

**Fig. 5:**
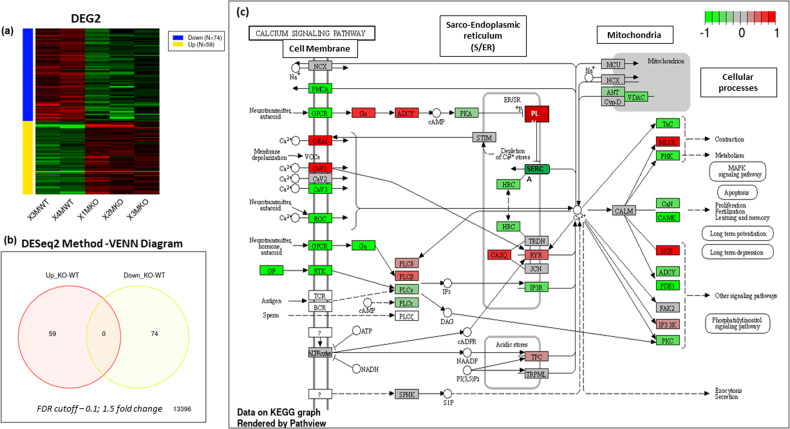
Summary of data obtained from DEG2 analysis differentially expressed genes in Brn-3b KO aortas when compared with WT controls. **a** Heat map showing differential expression of genes (DEG) in Brn-3b KO aortas, compared with WT controls- down-regulated = top panel; up-regulated = bottom panel. **b** VENN diagram showing the numbers of up and down-regulated genes, using FDR cut-off = 0.1; fold change =1.5 **c** Schematic diagram showing how genes that are up-regulated or down-regulated in Brn-3b KO aortas are linked in the calcium signalling pathway. Modified KEGG Image was rendered by Pathview with red boxes indicating genes that were increased in Brn-3b KO aortas while green boxes indicate decreased genes and grey indicate unchanged expression.

DEG2 was next undertaken to identify the most significantly up- and down regulated genes, upon loss of Brn-3b. DESeq analyses showed that 59 genes were significantly upregulated and 74 genes down-regulated in Brn-3b KO aorta (Fig. 5a, b). Selected genes shown in Table 2a, b, highlight how genes that were up- or down regulated upon loss of Brn-3b affected key pathways involved in Ca^2+^ signalling and muscle contraction and is summarised in Fig. 5c, which shows KEGG graph and proposed effects on calcium signalling pathways.

**Table 2:** Selected genes that were differentially regulated in Brn-3b KO aortas.

a) Selected Genes increased in male Brn-3bKO aorta			
Symbol	KO-WT log2 Fold Change	ENSEMBL	Description
***Calcium regulation***			
Ryr2	1.79	ENSMUSG00000021313	ryanodine receptor 2, cardiac
Cacna2d2	1.41	ENSMUSG00000010066	calcium channel, voltage-dependent, alpha 2/delta subunit 2
Cacna1h	1.83	ENSMUSG00000024112	calcium channel, voltage-dependent, T type, alpha 1H subunit
Cacna1d	1.04	ENSMUSG00000015968	calcium channel, voltage-dependent, L type, alpha 1D subunit
Pln	0.95	ENSMUSG00000038583	phospholamban
Camk1g	0.94	ENSMUSG00000016179	calcium/calmodulin-dependent protein kinase I gamma
***Muscle (Sarcomeric or regulatory proteins)***			
Pla2g2e	1.55	ENSMUSG00000028751	phospholipase A2, group IIE
Tcap	1.87	ENSMUSG00000007877	titin-cap
Nppa	1.78	ENSMUSG00000041616	natriuretic peptide type A
Mmp12	1.62	ENSMUSG00000049723	matrix metallopeptidase 12
***Other***			
Ciart	4.67	ENSMUSG00000038550	circadian associated repressor of transcription
Dbp	4.18	ENSMUSG00000059824	D site albumin promoter binding protein
Gpr22	3.13	ENSMUSG00000044067	G protein-coupled receptor 22
Alox8	2.47	ENSMUSG00000020891	arachidonate 8-lipoxygenase
Nr1d2	2.43	ENSMUSG00000021775	nuclear receptor subfamily 1, group D, member 2
Thsd7b	2.22	ENSMUSG00000042581	thrombospondin, type I, domain containing 7B
Hcn1	2.15	ENSMUSG00000021730	hyperpolarization activated cyclic nucleotide gated potassium channel 1
Nr1d1	1.75	ENSMUSG00000020889	nuclear receptor subfamily 1, group D, member 1
Cyp2f2	1.78	ENSMUSG00000052974	cytochrome P450, family 2, subfamily f, polypeptide 2
Per2	1.69	ENSMUSG00000055866	period circadian clock 2
Hamp	1.67	ENSMUSG00000050440	hepcidin antimicrobial peptide
Gpr158	1.66	ENSMUSG00000045967	G protein-coupled receptor 158
Kcp	1.56	ENSMUSG00000059022	kielin/chordin-like protein [
Slc16a12	1.56	ENSMUSG00000009378	solute carrier family 16 (monocarboxylic acid transporters), member 12
Zfp791	1.54	ENSMUSG00000074194	zinc finger protein 791
Wdfy1	1.44	ENSMUSG00000073643	WD repeat and FYVE domain containing 1

b) Selected genes down-regulated in male Brn-3b KO aorta			
***Calcium regulation***			
Atp2a1	−2.57	ENSMUSG00000030730	ATPase, Ca++ transporting, cardiac muscle, fast twitch 1
Tnni2	−2.38	ENSMUSG00000031097	troponin I, skeletal, fast 2
S100a9	−2.28	ENSMUSG00000056071	S100 calcium binding protein A9 (calgranulin B)
Ryr1	−2.19	ENSMUSG00000030592	ryanodine receptor 1, skeletal muscle
S100a8	−2.09	ENSMUSG00000056054	S100 calcium binding protein A8 (calgranulin A)
Trpm2	−1.38	ENSMUSG00000009292	transient receptor potential cation channel, subfamily M, member 2
Casq1	−0.95	ENSMUSG00000007122	calsequestrin 1
Orai2	−0.65	ENSMUSG00000039747	ORAI calcium release-activated calcium modulator 2
***Muscle (Sarcomeric or regulatory proteins)***			
Actn3	−2.64	ENSMUSG00000006457	actinin alpha 3
Mybpc2	−2.65	ENSMUSG00000038670	myosin binding protein C, fast-type
Mylpf	−2.58	ENSMUSG00000030672	myosin light chain, phosphorylatable,
Myh1	−2.18	ENSMUSG00000056328	myosin, heavy polypeptide 1, skeletal muscle,
Myl3	−1.90	ENSMUSG00000059741	myosin, light polypeptide 3
Eln	−1.86	ENSMUSG00000029675	elastin
Mmp8	−1.47	ENSMUSG00000005800	matrix metallopeptidase
Foxs1	−1.69	ENSMUSG00000074676	forkhead box S1
Ckm	−1.06	ENSMUSG00000030399	creatine kinase, muscle
Mylk2	−1.04	ENSMUSG00000027470	myosin, light polypeptide kinase 2
Lox	−1.08	ENSMUSG00000024529	lysyl oxidase
Gpr20	−1.01	ENSMUSG00000045281	G protein-coupled receptor 20
Npr1	−0.98	ENSMUSG00000027931	natriuretic peptide receptor 1
Mmp3	−0.94	ENSMUSG00000043613	matrix metallopeptidase 3
Adamts15	−0.94	ENSMUSG00000033453	disintegrin-like &metallopeptidase thrombospondin type 1 motif, 15
Angptl4	−0.88	ENSMUSG00000002289	angiopoietin-like 4
Mmp9	−0.88	ENSMUSG00000017737	matrix metallopeptidase 9
***Immune***			
Cxcr2	−2.99	ENSMUSG00000026180	chemokine (C-X-C motif) receptor 2
Serpina3k	−2.78	ENSMUSG00000058207	serine (or cysteine) peptidase inhibitor, clade A, member 3K
Il1b	−2.38	ENSMUSG00000027398	interleukin 1 beta
Ccl12	−1.54	ENSMUSG00000035352	chemokine (C-C motif) ligand 12
Nfil3	−1.46	ENSMUSG00000056749	nuclear factor, interleukin 3, regulated
Trim10	−1.39	ENSMUSG00000073400	tripartite motif-containing 10
Csf3r	−1.68	ENSMUSG00000028859	colony stimulating factor 3 receptor (granulocyte)
Igsf9	−1.25	ENSMUSG00000037995	immunoglobulin superfamily, member 9
***Others***			
Arntl	−2.51	ENSMUSG00000055116	aryl hydrocarbon receptor nuclear translocator-like
Cyp1a1	−1.71	ENSMUSG00000032315	cytochrome P450, family 1, subfamily a, polypeptide 1
Rnf150	−1.67	ENSMUSG00000047747	ring finger protein 150
Dhrs9	−1.44	ENSMUSG00000027068	dehydrogenase/reductase (SDR family) member 9
Cdkn1a	−1.43	ENSMUSG00000023067	cyclin-dependent kinase inhibitor 1A (P21)
Slc25a34	−1.37	ENSMUSG00000040740	solute carrier family 25, member 34
Padi4	−1.18	ENSMUSG00000025330	peptidyl arginine deiminase, type IV
Retn	−0.67	ENSMUSG00000012705	resistin

a) Selected upregulated genes that in male Brn-3b KO aortas are grouped according to key known function; log-2-fold change [with (+) indicating upregulation and (−) downregulation]; ENSEMBL ID and brief description of each gene.

b) List of selected genes that were down-regulated in male Brn-3b KO aortas.

**Fig. 6:**
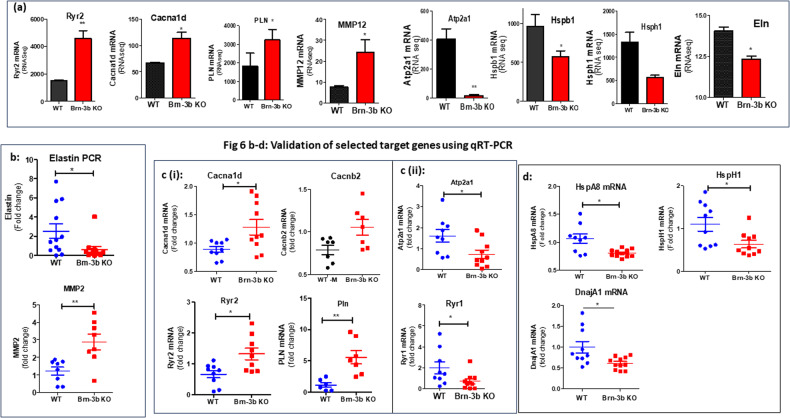
Differentially regulated genes from RNA sequencing data and qRT-PCR validation of selected genes. **a** Selected genes showing differential expression in Brn-3b KO aortas (*n* = 3) compared with WT aortas (*n* = 2) in RNA sequencing analysis. **b** qRT-PCR validation of selected genes associated with structural vascular changes including reduced elastin and increased MMP 2. **c** qRT-PCR validation of genes involved in calcium signalling pathway that were either (i) upregulated or (ii) down regulated in Brn-3b KO mutants when compared with WT controls. **d** Validated genes encoding chaperone proteins, involved in protection against stress, which were down regulated in Brn-3b KO mutants when compared with WT controls. Variation between RNA samples was adjusted using 36B4 or GAPDH and values are expressed as fold changes relative to reference samples included in each experiment. Data represent mean (±sd) from *n* = 8–10 independent samples with statistical analysis undertaken using students *t* test (*p < 0.05).

Notable changes in Ca^2+^ signalling gene included up-regulation of CaV channels Cacn2d2, Cacna1d & Cacna1h but also RyR2 and Pln [62, 63] with reduced Atp2a1, S100e9 (Calgranulin A), and RyR1. Similarly, changes were detected in genes encoding sarcomeric proteins that affected muscle structure and VSMC contractility. These changes included up-regulation of Mmp12 (Table 2a; Fig. 6a) but also calcium-dependent phospholipase A2, (Pla2g2e) and atrial natriuretic peptide A (Nppa) while there was significant reduction of Eln (Table 2b; Fig. 6a) and Actn3; Mylpf; Myl3. Other upregulated genes were linked to control of circadian processes or metabolic function while many down regulated genes including Cxcr2, IL1 beta, Ccl12, Csf3r and Igsf9 are implicated in the control of immune responses that may indirectly affect vascular function [64–67]. These results suggest that Brn-3b can regulate vascular contractility by activating or repressing genes that control Ca^2+^ signalling and muscle contractility either directly or indirectly via other processes such as inflammation or metabolic changes. Therefore deregulated expression of such genes upon loss of Brn-3b will contribute to abnormal vascular phenotype and contractile function (s-Fig. 3).

### Validation of selected genes

Since loss of Brn-3b caused significant changes in Ca^2+^ signalling genes and S/ER function, these pathways were used as the basis for validation studies that were focused on selected genes in each pathway. Figure 6b–d shows the results of qRT-PCR analysis of selected genes using cDNA from independent Brn-3b KO or WT aortas. For instance, Fig. 6b shows significant reduction in the gene encoding elastin in Brn-3b KO aortas but also increased MMP2 expression, which is associated with elastin degradation. Figure 6c (i) shows selected genes involved in Ca^2+^ signalling that were significantly increased in Brn-3b KO aortas, including CaV channel Cacna1d and S/ER Ca^2+^ channel RyR2 but also PLN, which inhibits S/ER Ca^2+^ pump. Similar but non-significant increases were also seen for Cacnb2. In contrast, Fig. 6c (ii) confirms marked reduction in ATP2a encoding SERCA1 Ca^2+^ pump (required for Ca^2+^ reuptake into the S/ER) and Ryr1 in Brn-3b KO mutant aortas when compared with WT controls. Interestingly, qRT-PCR data also confirmed statistically significant reduction in genes encoding chaperone proteins such as HspA8, HspH1 and DnaJa1, that are required for UPR in S/ER (Fig. 6d). Since reduced S/ER Ca^2+^ reuptake has been implicated in increasing S/ER stress (Supplementary Fig. 3), reduced expression of chaperone proteins also suggest increased ER stress that can affect VSMC phenotype and function, thereby contribute to vascular dysfunction in Brn-3b KO aortas [68].

### Abnormal calcium signalling in VSMC cultured from Brn-3b KO aortas

**Fig. 7:**
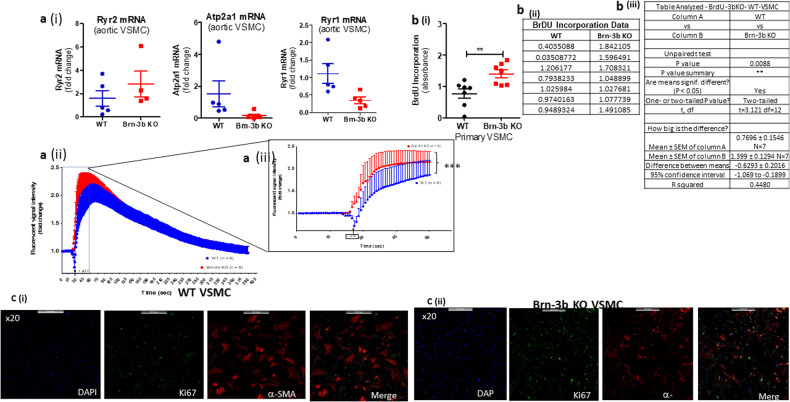
Attenuated calcium release responses of Brn-3b KO VSMC is linked to increased proliferative rate. **a** (i) qRT-PCR data showing changes in mRNA encoding selected genes (Ryr2, Atp2a1, and Ryr1) in total RNA prepared from primary VSMC cultures that were generated using WT or Brn-3b KO aortas (*n* = 4–5 aortas per group). (ii) Graph showing quantification of Ca^2+^ transients in primary VSMC cultures from WT or Brn-3b KO aortas, following treatment with 100 µmol ATP. The data shows changes in fluorescent signal intensity over time following treatment with ATP in either WT VSMCs (blue) or Brn-3b KO VSMCs (red). Signals were obtained using confocal microscopy followed by image J analysis with data representing values from six independent WT and Brn-3b KO aortas. (iii) Detailed images showing the significant changes in signal intensity in Brn-3b KO VSMCs, within the first 30 s after ATP treatment, when compared with matched WT controls. To highlight the statistical differences, which was determined using two-way ANOVA. **b** (i) Graph showing data from BrdU incorporation studies to analyse rate of change in DNA synthesis in primary VSMC cultures from WT or Brn-3b KO aortas. Data represents the mean and standard error of values obtained from seven independent VSMC cultures. (ii) table showing data from independent samples from WT or Brn-3b KO VSMC cultures. (iii) Statistical analysis undertaken using students *t* test shows significant differences in VSMCs from WT and Brn-3b KO aortas. **c** Representative fluorescent images of VSMC cultures prepared from either WT or Brn-3b KO aortas and stained with Ki-67 Ab (green) or α-SMA (red). DAPI staining (blue) was used to identify the cell nuclei and merge samples show the overlay of the images as different wavelengths.

To determine if similar gene expression changes were observed in primary VSMC cultures from mutant mice, qRT-PCR were also undertaken to analyse for expression changes in selected Ca^2+^ signalling genes that were altered in Brn-3b KO aortas. Results shown in Fig. 7a(i) demonstrate that VSMC cultures prepared from Brn-3b KO aortas showed similar changes in Ryr2, Atp2a1 and Ryr1 when compared with WT controls, thereby confirming that such gene expression changes were likely to be linked to VSMC in the aorta.

Therefore, we next tested if deregulation in genes required for /ER Ca^2+^ homoeostasis in Brn-3b KO mutants affected Ca^2+^-mediated contractile responses in VSMC by analysing Ca^2+^ handling in isolated primary VSMCs from mutant and WT aortas.

For these studies, baseline signals were recorded from sensitised VSMC cultures prepared from Brn-3b KO or WT aortas and loaded with fluo-4-AM dye. Changes in signal intensity was recorded over time following treatment with ATP (1 um) (0–400 s). Figure 7a(ii) shows a summary of changes in Ca^2+^ flux over time, from ≥6 independent cultures, which indicate that ATP treatment triggered higher peak intensities in Ca^2+^ transients in Brn-3b KO VSMCs, when compared with WT controls. The most significant changes were evident within the first 30 s after ATP treatment [Fig. 7a(iii)] (*p* value < 0.0001 in two-way ANOVA), suggesting rapid increase in S/ER Ca^2+^ release in Brn-3b KO aortas, which may be linked to increased RyR2 levels. Taken together, these results confirmed that genes affected by loss of Brn-3b can alter Ca^2+^ homoeostasis in VSMCs and thereby affect aortic contractility.

### Increased proliferation in VSMC from Brn-3b KO aortas

Increased intracellular Ca^2+^ can also trigger phenotypic switching of VSMC by activating NFAT mediated gene transcription, thereby promoting proliferation [14]. Therefore, to determine if increase calcium transients affected the proliferative index of Brn-3bKO VSMC BrdU incorporation experiments were undertaken to measure the rates of DNA synthesis in proliferating cells in WT and KO cultures. Figure 7b shows the pooled results from seven independent experiments which clearly demonstrates increased proliferation in VSMC cultures from Brn-3b KO aortas when compared with WT cultures.

To confirm changes in the rates of proliferation, VSMCs cultures from Brn-3b KO and WT aortas were co-immunostained for Ki-67 antigen, which acts as a marker of cell proliferation and α-SMA to mark VSMCs. Representative immunostaining images shown in Fig. 7c, demonstrates larger number of Ki-67 positive cells (green) in cultures from Brn-3b KO aortas, when compared with WT controls. Interestingly, α-SMA staining, which is a marker of contractile VSMCs showed significantly higher intensity staining in WT cultures when compared with Brn-3b KO cells. This may suggest that increased proliferation in Brn-3b KO derived VSMC may be accompanied by reduced numbers of differentiated VSMCs in Brn-3b KO cultures.

These results, taken together with increased ECM synthesis in Brn-3b KO mutants indicate that loss of Brn-3b may contribute to phenotypic switching in VSMC and thereby affect vascular contractility and function.

**Fig. 8:**
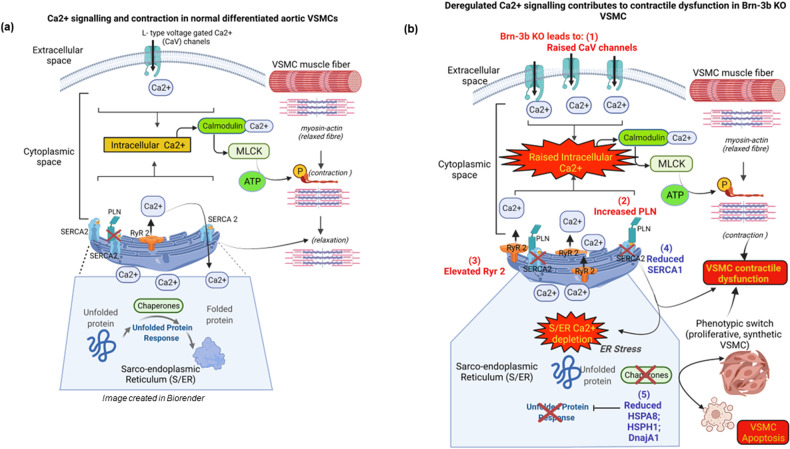
Proposed mechanism by which gene expression changes caused by loss of Brn-3b may affect VSMC fate and function and thereby vascular contractility. **a** Schematic diagram of Ca2+ regulation of contraction and S/ER stress responses in normal contractile VSMC. **b** Effects caused by loss of Brn-3b which caused changes in selected genes that regulate Ca2+ signalling or stress responses in Brn-3b KO VSMC. Genes up-regulated in Brn-3b KO cells are shown in red and down-regulated genes in blue. **‘**X’ indicates inhibition of function or loss of expression. *Image created in by Biorender*.

These results have led us to a proposed model whereby Brn-3b acts as an important regulator of key target genes that are required for maintaining normal vascular function. This may occur via different effects including regulation of structural components (e.g. elastin and collagen) or essential pathways that control Ca^2+^ signalling and S/ER stress responses. Therefore, loss of Brn-3b intracellular levels will lead to increased intracellular Ca^2+^ and S/ER Ca^2+^ depletion but also increase ER stress and thereby lead to phenotypic switching and contractile dysfunction in VSMC’s (Fig. 8).

## Discussion

VSMCs are essential for active regulation of arterial compliance and vascular tone in vessels, such as the aorta since changes in the phenotype and function of these cells can contribute significantly to the development of vascular dysfunction e.g. arterial stiffening & hypertension and increase the risk of CVD [8, 9, 13, 14] [69, 70]. Yet, the chronic nature of such diseases means that such vascular damage often develop over relatively long periods but often remain asymptomatic and undetectable at early stages so the underlying molecular basis of such diseases have remained poorly understood, thereby frustrating attempts at early diagnosis and effective treatment to prevent irreversible damage [9, 12].

In this study, we showed for the first time that Brn-3b has essential but previously unknown roles in controlling vascular integrity and function by regulating the expression of genes that control Ca^2+^ signalling and contractile function as well as stress responses in VSMCs. While the first evidence pointing to Brn-3b expression in the vasculature arose from studies in stressed hearts showing that loss of Brn-3b caused extensive adverse remodelling around the coronary vasculature in male Brn-3b KO mice [26], the current studies has confirmed that the shorter isoform, Brn-3b(s), was the main Brn-3b protein expressed in the aorta. Co-immunostaining studies also confirmed expression of the Brn-3b protein in BV walls, with localisation in VSMC seem either in aortic sections or isolated primary VSMC cultures. The aorta provided a useful model to analyse Brn-3b expression in the vasculature but was also relevant because adverse changes in aortic elastance and compliance can affect cardiac contractility and contribute to abnormal contractile responses, previously reported in 2mth old Brn-3b KO mutants [26]. Moreover, the aorta provided a valuable source for isolating and analysing primary VSMC using standard in vitro culture.

The availability of the constitutive Brn-3b KO mutants also provided insights into the effects of loss of Brn-3b on aortic phenotype and function. The current studies were primarily focused on male mutants because of previous data showing that male Brn-3b KO mice developed extensive coronary artery remodelling following chronic stress, which was not seen in female mutants [26]. As such, structural and histological abnormalities in aortas from male Brn-3b KO mutants were particularly interesting. For example, small but consistent narrowing/coarctation defects in the descending aortas from 2mth old male Brn-3b KO mice were similar to abnormalities previously shown in heterozygote Eln^+/−^ mice, where reduced elastin was strongly associated with hypertension [71, 72]. In line with this, histological staining of aortic sections, which showed disruption of elastin fibres seen in Brn-3b KO aortas were supported by ultrastructural changes observed in TEM images. Interestingly, RNA sequencing data showed that not only was Eln mRNA reduced in Brn-3b KO aortas, but mutant aortas also expressed higher levels of MMP12 elastase, which can contribute to disruption of elastin fibres seen in TEM images. TEM and immunostaining also confirmed increased collagen deposition in the thickened adventitial layers of Brn-3b KO aortas, which when combined with the observed reduction in lumen diameter can also contribute to arterial stiffening and contractile dysfunction [73]. This was indeed confirmed by wire myography with differences in IC100 in vascular rings from Brn-3b KO aortas potentially link to reduced lumen diameter and/or pre-contraction of vessels at baseline. In addition, abnormal responses in mutant BV was also evident from responses to treatments with known contractile stimuli. This included α-adrenergic agonist, PE, which elicited strong phasic and tonic contraction in WT VSMC via CaV channels [52, 74] but caused markedly attenuated responses in vascular rings from Brn-3b KO aortas. Similarly, while prostaglandin analogue U46619 and KCl induced strong contraction in WT VSMCs by raising intracellular Ca^2+^ levels via G-protein coupled receptors or via S/ER channels [49, 75] [52, 76] similar treatment elicited minimal responses in vascular rings from Brn-3b KO aortas. Importantly, vascular contractility induced by such treatment are mediated by effects of the Ca^2+^ signalling pathways [11, 77]^,^. This is particularly important in VSMC, where active contraction is tightly regulated by changes in intracellular Ca^2+^, with contraction controlled by expression and activity of specialised Ca^2+^ channels either in the plasma membrane or S/ER, which serve to increase intracellular Ca2+ and initiate vasoconstriction [13, 54, 78]. Conversely, repolarisation and relaxation depend upon rapid reduction of intracellular Ca^2+^ either by specialised pumps such as PMCA, which promote Ca^2+^ efflux or SERCA pumps, which facilitate Ca^2+^ reuptake into S/ER intracellular stores [10, 12, 76]. Therefore, abnormal contractile responses in vascular rings from Brn-3b KO aortas suggest deregulation of Ca^2+^ signalling pathways and in line with this, data from high throughput RNA sequencing showed that Ca^2+^ signalling genes were most significantly affected by loss of Brn-3b, suggesting that loss of Brn-3b can profoundly altered Ca^2+^ signalling pathways in VSMCs.

Indeed, K-means and KEGG pathway analysis showed that the largest numbers of genes affected by loss of Brn-3b were associated with Ca^2+^ signalling and vascular contraction while GO analysis confirmed that these genes encoded for proteins associated with plasma membrane or S/ER complexes that were involved in biological processes including Ca^2+^ and cation transport or muscle contraction [53]. Since Ca^2+^signalling also controls contractility in cardiomyocytes, it is perhaps unsurprising that cardiac dysfunction and cardiomyopathies were also identified as affected pathways [79]. Similarly, GSEA and DEG analysis showed that the largest number of upregulated genes (158) in Brn-3b KO aortas were implicated in Ca^2+^ signalling pathway. On the other hand, 93 upregulated genes and 68 down-regulated genes were associated with VSMC contraction and associated with key pathways including actin cytoskeleton and Apelin signalling pathways, which controls vasodilation and, importantly, are reduced in experimental hypertension models but also hypertensive patients [61, 80–82]. The link between loss of Brn-3b and hypertension was further reinforced by Jensen.Disease pathway analysis of RNA sequencing data which identified hypertension as the top disease pathway affected in Brn-3b KO mutant aortas involving 169 upregulated genes while 120 affected genes were associated with CAD in humans. This was particularly interesting because of existing GWAS data showing that SNPs within the Brn-3b genomic locus (chromosome 4q.31.2) were strongly associated with CHD, CAD and CVA [40–45]. These data indicate that changes in Brn-3b expression in the vasculature can contribute to contractile dysfunction and CVD.

Subsequent validation studies confirming that genes involved in of Ca^2+^ signalling and S/ER pathways were significantly and reproducibly de-regulated in independent Brn-3b KO aortas and primary Brn-3b KO VSMC cultures, were particularly important for identifying the underlying mechanisms that drive contractile abnormalities in Brn-3b KO mutants [83, 84].

For instance, raised intracellular Ca^2+^ in VSMC, which can occur either by Ca^2+^ influx from extracellular space following activation of activation of Ca^2+^ channels e.g. L-type voltage-gated Ca^2+^ (CaV) in the plasma membrane or by Ca^2+^ release from intracellular store e.g. S/ER via inositol triphosphate (IP_3_) receptors or via ryanodine receptors (RyR), which contributes to localised Ca^2+^increases (Ca^2+^ transient or sparks) [83, 85]. Increased intracellular Ca^2+^ can then triggers VSMC contraction by driving calmodulin mediated activation of myosin light chain kinase and phosphorylation of myosin light chain (MLC), resulting in actin–myosin interaction [10, 86].

Conversely, membrane re-polarisation and VSMC relaxation depends on reduction of intracellular Ca^2+^ levels by its re-uptake into intracellular stores by S/ER Ca^2+^ATPase (SERCA) pumps (encoded by ATP2a genes) [68], or by efflux via plasma membrane Ca^2+^ (PMCA) pumps, encoded by ATP2b genes [13, 15, 69]. Ca^2+^ reuptake into the S/ER is also affected by regulatory proteins such as PLN which inhibits SERCA pump activity. Therefore, changes in genes regulating intracellular Ca^2+^ levels can cause contractile dysfunction in VSMCs [13, 15, 69].

In this regard, increased expression of genes encoding long-acting L‐type CaV channels e.g. Cacna1d and Cacnb2, in Brn-3b KO aortas will raise intracellular Ca^2+^ and trigger contraction in VSMCs by facilitating Ca^2+^ influx via plasma membrane. Therefore, such Ca^2+^ channels are often targeted by Ca^2+^ channel blockers used to treat patients with hypertension [63, 76, 84]. Similarly S/ER Ca^2+^ channels are also affected. For instance, while RyR1 is reduced, increased expression of the RyR2 channels in Brn-3b KO aortas will also promote release of Ca^2+^ from the S/ER into the cytoplasm to increase intracellular Ca^2+^ while inducing localised transients that also contribute to vascular contraction [13, 83, 85]. As such, increases in these Ca^2+^ channel genes in Brn-3b KO VSMCs can promote abnormal vascular contractility both at baseline and in response to stimulus, as observed in myography [62, 87]. Moreover, sustained increases in intracellular Ca^2+^ may also contribute to phenotypic switching from contractile into proliferative VSMCs because raised intracellular Ca^2+^ can activate NFAT TF (via calcineurin pathway) to drive transcription of genes associated with cell proliferation [74].

On the other hand, loss of Brn-3b may also affect VSMC re-polarisation and relaxation in the aortas because although Atp2a1 was reduced and Atp2a2 levels remain unchanged, increased expression of the potent SERCA pump inhibitor, Pln, will prevent Ca^2+^ reuptake into the S/ER. Inhibition of SERCA activity combined with enhanced Ca^2+^ release (via increased Ryr2) will lead to S/ER Ca^2+^ depletion and contribute to ER stress and UPR [19]. However, reduction in chaperone proteins such as the known Brn-3b target gene Hsp27 (HspB1) [29] as well as other chaperone genes including HspA8, HspH1 and DnaJa1, will prevent adaptive UPR and thereby trigger phenotypic switching from contractile into proliferative VSMCs [20, 69]. This was indeed confirmed in studies using primary VSMC cultures from Brn-3b KO aortas, which displayed significant hyperresponsiveness to ATP stimulation, with increases peak intensity of Ca^2+^ transients during early stages reflecting increased Ca^2+^ release from S/ER stores via IP3R and RyR [69].

Such abnormal Ca^2+^ responses combined with increased proliferation rates in Brn-3b KO VSMC suggests that deregulated intracellular Ca^2+^ may help to contribute to phenotypic switching of contractile VSMCs into proliferative, synthetic VSMCs [88]. Whilst additional studies will be needed to confirm whether phenotypic switching occurs upon loss of Brn-3b, it is known that increased intracellular Ca^2+^ can lead to activation of the NFAT TF (via calcineurin pathway), which in turn, activates transcription of genes associated with cell proliferation. Therefore, deregulation of genes involved in Ca^2+^ signalling, which are caused by loss of Brn-3b, may also contribute to phenotypic and functional changes in the aortic VSMCs and supports important roles for Brn-3b in controlling vascular integrity and function.

These results suggest that Brn-3b is important for regulating genes that control Ca^2+^ signalling and VSMC contraction in arterial blood vessels and loss of Brn-3b will contribute to contractile dysfunction, which precedes the development of vascular dysfunction including hypertension and subsequent progression to CAD. Therefore, elucidating the effects of loss of Brn-3b and its target genes could provide insight into the molecular basis of early vascular changes and subsequent development vascular dysfunction /damage [89].

Since this study was undertaken using the constitutive Brn-3b KO mutants, it is noteworthy that loss of Brn-3b caused deregulation of genes that are implicated in regulating immune responses, circadian entrainment and metabolic processes, which can indirectly affect vascular function. Although future studies using tissue-specific mutants can help to determine direct and indirect effects of loss of Brn-3b in the vasculature, changes in different subsets of genes are nonetheless interesting and relevant in view of previous studies showing Brn-3b expression and effects of its loss on relevant tissues. For instance, previous studies showing metabolic dysfunction in Brn-3b KO mutants (hyperglycaemia, insulin resistance and increased visceral WAT deposits [26, 30, 35]), means that deregulation of metabolic genes in Brn-3b KO aortas was unsurprising. However, despite careful attempts to remove all excess tissues around the aortas used for RNA sequencing, at this stage it is unclear if such genes are affected by metabolic changes in the mutant aortas or are link to residual PVAT. Nevertheless, these observations are also interesting because adipose tissues can exert profound endocrine effects on BV wall by producing vasoactive modulators such as adipokines and cytokines [90–92]. Indeed, common metabolic dysfunction such as obesity and diabetes are commonly linked to increased risk factors of damage to coronary and systemic circulation and subsequent CVD [12]. Since Brn-3b KO PVAT display significant increases in WAT content which produces pro-inflammatory adipokines and cytokines, this is likely to affect the inflammatory milieu and thereby indirectly alter VSMC phenotype and function [60, 90, 93, 94]. In line with this, TEM images showed infiltration of inflammatory cells into the PVAT surrounding Brn-3b KO aortas not seen in PVAT in WT aortas. Inflammatory changes were also confirmed by RNA sequencing data, which showed significant changes in genes linked to pro-inflammatory pathways e.g. IL-17 signalling and cytokine-cytokine receptor interaction pathways, and are implicated in development of vascular dysfunction and CVDs including hypertension and atherosclerosis [2]. Although the link between loss of Brn-3b and deregulated immune responses are still to be determined, Brn-3b is expressed in immune cells e.g. monocytes [34, 95], T cells and PBMCs [96, 97], so it will be interesting to determine if inflammatory effects in Brn-3b KO tissues cause direct or indirect effects on vascular function and transition to vascular diseases [58]. Furthermore, the implications and effects of upregulation of genes that control circadian processes changes in Brn-3b KO aortas remain unknown but since disruption of the circadian rhythm is commonly associated with ageing and risk of vascular, cardiac and metabolic diseases [58, 98, 99], these mutants model could provide some interesting insight into the mechanisms linking such conditions.

In conclusion, the Brn-3b TF represents a novel and important regulator of genes that control Ca^2+^ in VSMCs and thereby regulate vascular contractility and function. Since Ca^2+^ signalling pathways are pivotal for controlling multiple cellular functions in different cell types, regulation of such genes by Brn-3b could have wider implications in understanding how this transcriptional regulator controls gene expression and cell fate in other cellular contexts in which Ca^2+^ pathways are also critical for function. Loss of Brn-3b also appears to be linked to structural and functional changes in the aortas and was strongly linked to hypertension and coronary artery disease either in Jensen.Disease pathway analysis or GWAS data showing that SNPs in Brn-3b genomic region (chromosome 4q31) were associated with increased risk of hypertension and CHD [40–42]. Therefore, these novel findings may provide insight into if/how reduction or loss of Brn-3b may drive early vascular changes during early stages of vascular dysfunction and progression to disease. Finally, deregulation of genes involved in other key processes including immune function, circadian regulation and metabolic processes in Brn-3b KO aortas points to complex but as yet unknown mechanisms by which this regulator may indirectly affect vascular function.

### Supplementary information

Original Data File

S-Fig 1

Supplementary Table 1

Supplementary Table 2

reprorting summary

## Data Availability

RNA sequencing datasets generated and analysed during the current study are submitted as fastq files in National Centre for Biotechnology Information (NCBI) sequence read archive (SRA) database repository (https://www.ncbi.nlm.nih.gov/sra) as PRJNA1034077, after release date 2024-05-01. Gene counts used for iDEP analysis are also available from the corresponding author, on reasonable request.
