# Supplementary material for: Vascular dysfunction caused by loss of Brn-3b/POU4F2 transcription factor in aortic vascular smooth muscle cells is linked to deregulation of calcium signalling pathways
Source: Cell Death Dis. 2023 Nov 25;14(11):770. doi: 10.1038/s41419-023-06306-w (PMC10676411; doi:10.1038/s41419-023-06306-w)
Supplement: Supplementary file 1 — Original Data File [file 41419_2023_6306_MOESM1_ESM.pptx]

## Slide 1
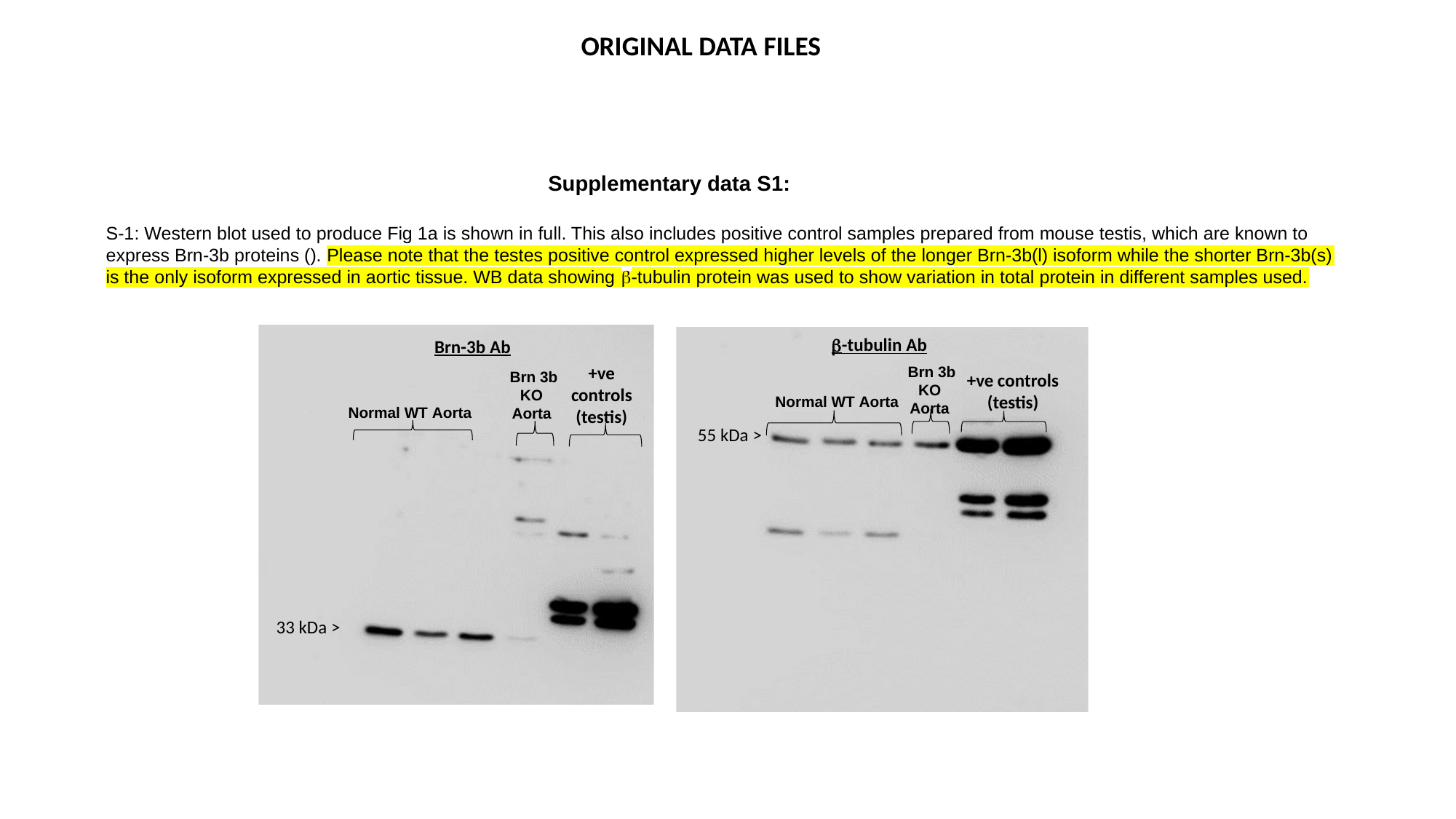

ORIGINAL DATA FILES
Supplementary data S1:
S-1: Western blot used to produce Fig 1a is shown in full. This also includes positive control samples prepared from mouse testis, which are known to express Brn-3b proteins (). Please note that the testes positive control expressed higher levels of the longer Brn-3b(l) isoform while the shorter Brn-3b(s) is the only isoform expressed in aortic tissue. WB data showing b-tubulin protein was used to show variation in total protein in different samples used.
b-tubulin Ab
Brn 3b
KO
Aorta
+ve controls (testis)
Normal WT Aorta
 55 kDa >
Brn-3b Ab
Brn 3b
KO
Aorta
Normal WT Aorta
 33 kDa >
+ve controls (testis)
