# Supplementary material for: Vascular dysfunction caused by loss of Brn-3b/POU4F2 transcription factor in aortic vascular smooth muscle cells is linked to deregulation of calcium signalling pathways
Source: Cell Death Dis. 2023 Nov 25;14(11):770. doi: 10.1038/s41419-023-06306-w (PMC10676411; doi:10.1038/s41419-023-06306-w)

Supplementary data S1:

S-1: Western blot used to produce Fig 1a is shown in full. This also includes positive control samples prepared from mouse testis, which are known to express Brn-3b proteins. Please note that the testes positive control expressed higher levels of the longer Brn-3b(l) isoform while the shorter Brn-3b(s) is the only isoform expressed in aortic tissue. WB data showing β-tubulin protein was used to show variation in total protein in different samples used.

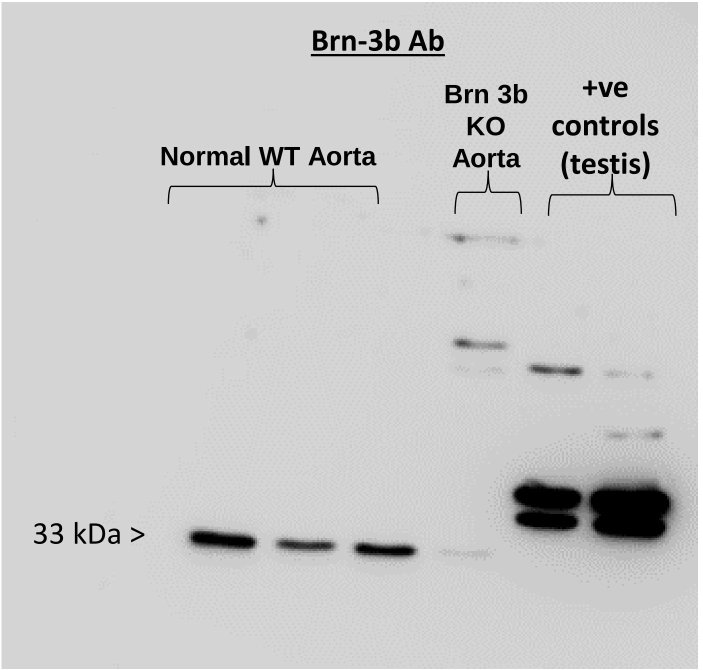

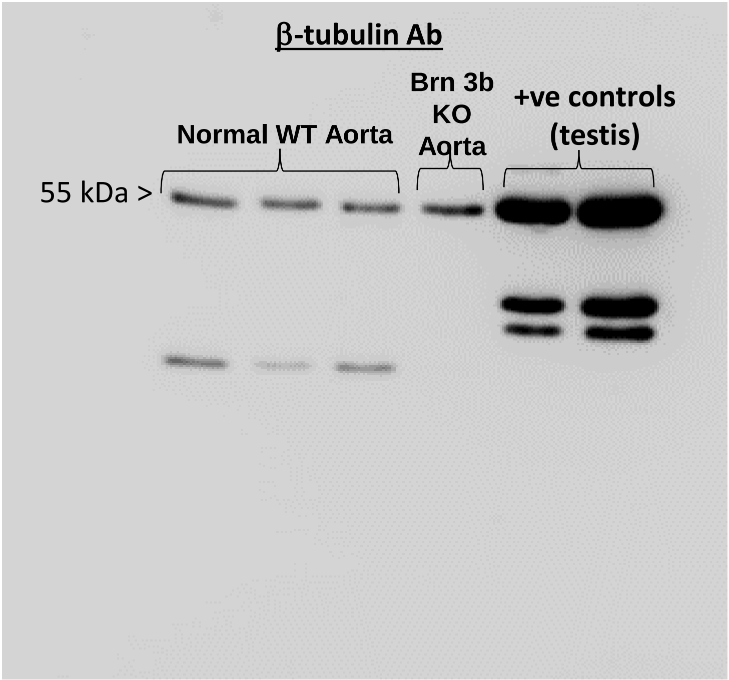

Supplement: Supplementary file 2 — S-Fig 1 [file 41419_2023_6306_MOESM2_ESM.pdf]
