# Supplementary material for: Vascular dysfunction caused by loss of Brn-3b/POU4F2 transcription factor in aortic vascular smooth muscle cells is linked to deregulation of calcium signalling pathways
Source: Cell Death Dis. 2023 Nov 25;14(11):770. doi: 10.1038/s41419-023-06306-w (PMC10676411; doi:10.1038/s41419-023-06306-w)
Supplement: Supplementary file 3 — Supplementary Table 1 [file 41419_2023_6306_MOESM3_ESM.docx]

**Supplementary data – Table 1**

**S-Table 1:(a)** List of genes upregulated in Brn-3b KO aortas that are linked to muscle contractility and/or Ca2+ signalling. Arranged by number of genes, with significance indicated by normalisation enrichment scores (NES) and adjusted p-value.

| **Direction** | **Pathways** | **NES** | **nGenes** | **adj.Pval** | **Genes** |
| --- | --- | --- | --- | --- | --- |
| **Calcium signalling/muscle function:** | | | | | |
| Up | Regulation of muscle contraction | 1.72 | 111 | 0.00033 | Casq1 Anxa6 Fkbp1b Pde4d Casq2 Atp2a2 Dmpk Chrm2 Adrb2 Adra1a Chrm3 Tnnc1 Flna Ank2 Tacr2 Edn1 Tbxa2r Tnni3 Agrn Zc3h12a Scn4a Jup Mybpc3 Sri Kit Atp1a2 Rhoa Kcnq1 Stc1 Itga2 Srf Ccn2 Myocd Ryr2 Gata4 Dsc2 Tnnt2 Mylk2 Slc9a1 P2rx4 Atp2a1 Cttn Tnni2 Hcn4 Ptgs2 Rangrf Atp1a1 Adra2a Scn10a Kcne3 Dock4 Hspb6 Trpm4 Akap9 Nppa Kcnj2 Pkp2 Adora1 Dsg2 Dock5 Adra2c Kcna1 Oxtr Adra1b Cacna1c Myh7 Gper1 Slc8a1 Dsp Ptafr Gja5 Abat Rnf207 Calcrl Myl3 Ctnna3 Sphk1 Cav3 Tnnt1 Grcc10 Tmem38b Tmem38a Mrvi1 Actn3 Cav1 Nkx2-5 Ada Atp2b1 Smtn P2rx1 Dlg1 Sod1 Ehd3 Bin1 Smad7 Sumo1 Hdac4 Rgs2 Atp1b1 Pde4b Fgf13 Scn5a Chrnb4 Pln Npnt Dmd Arhgap42 Prkg1 Pde5a Setd3 Bscl2 Slc8a3 Lck Map2k1 Dbn1 Adrb1 Pawr Fxyd1 Ptger4 Tnni3k Shc1 Ptgs1 F2r Akap6 Cald1 Ryr1 |
| Up | Regulation of striated muscle contraction | 1.74 | 70 | 0.00033 | Casq1 Fkbp1b Pde4d Casq2 Atp2a2 Dmpk Flna Ank2 Agrn Zc3h12a Scn4a Jup Mybpc3 Sri Atp1a2 Kcnq1 Stc1 Ccn2 Ryr2 Gata4 Dsc2 Slc9a1 P2rx4 Atp2a1 Hcn4 Rangrf Atp1a1 Scn10a Kcne3 Tnni3 Trpm4 Akap9 Nppa Kcnj2 Pkp2 Adora1 Dsg2 Adra1a Adra1b Cacna1c Myh7 Slc8a1 Dsp Gja5 Rnf207 Myl3 Ctnna3 Cav3 Grcc10 Tmem38b Tmem38a Actn3 Cav1 Nkx2-5 Smtn Dlg1 Ehd3 Bin1 Smad7 Sumo1 Hdac4 Rgs2 Atp1b1 Pde4b Fgf13 Scn5a Pln Dmd Pde5a Bscl2 Slc8a3 Mylk2 Adrb1 Fxyd1 Tnni3k Akap6 |
| Up | Muscle organ morphogenesis | 1.68 | 65 | 0.00033 | Myom1 Obsl1 Mylk2 Myom2 Myom3 Mybpc2 Ttn Mybpc3 Tcap Nkx2-5 Zfpm2 Mylk Tnnt2 Tpm1 Sirt6 Tnni3 Myh6 Pkp2 Myh7 Myl3 Tnnc1 Hey2 Vangl2 Ccm2l Isl1 Fzd1 Foxc2 Fzd2 Foxc1 Heg1 Tgfb1 Tgfbr1 Prox1 Wnt2 Rxra Med1 Arid5b Adarb1 Gsc Ryr2 Ptcd2 Bmpr1a Wnt5a Smad4 Ankrd1 Smad7 Col3a1 Eng Notch1 Xirp2 Lrp2 Dll4 Bmp2 Serp1 Shox2 Pitx2 Ube4b Tbx20 Egln1 Fkbp1a Lif Rbpj Tgfb2 Chd7 S1pr1 Zfpm1 Dsp Tcf15 Actc1 Epor Ctnnb1 Gata4 Angpt1 |
| Up | Muscle tissue morphogenesis | 1.68 | 61 | 0.00033 | Myom1 Obsl1 Mylk2 Myom2 Myom3 Mybpc2 Ttn Mybpc3 Tcap Nkx2-5 Zfpm2 Mylk Tnnt2 Tpm1 Sirt6 Tnni3 Myh6 Pkp2 Myh7 Myl3 Tnnc1 Hey2 Vangl2 Ccm2l Isl1 Fzd1 Foxc2 Fzd2 Foxc1 Heg1 Tgfb1 Tgfbr1 Prox1 Wnt2 Rxra Med1 Adarb1 Ryr2 Ptcd2 Bmpr1a Wnt5a Smad4 Ankrd1 Smad7 Col3a1 Eng Notch1 Xirp2 Lrp2 Dll4 Bmp2 Shox2 Pitx2 Ube4b Tbx20 Egln1 Fkbp1a Rbpj Tgfb2 Chd7 S1pr1 Zfpm1 Dsp Actc1 Epor Ctnnb1 Angpt1 |
| Up | Cardiac muscle tissue morphogenesis | 1.67 | 55 | 0.00057 | Myom1 Obsl1 Mylk2 Myom2 Myom3 Mybpc2 Ttn Mybpc3 Tcap Nkx2-5 Zfpm2 Tnnt2 Tpm1 Sirt6 Tnni3 Myh6 Pkp2 Myh7 Myl3 Tnnc1 Hey2 Ccm2l Isl1 Foxc2 Foxc1 Heg1 Tgfb1 Tgfbr1 Prox1 Wnt2 Rxra Med1 Ryr2 Ptcd2 Bmpr1a Wnt5a Smad4 Ankrd1 Smad7 Eng Notch1 Xirp2 Lrp2 Dll4 Bmp2 Pitx2 Ube4b Tbx20 Egln1 Fkbp1a Rbpj Tgfb2 Chd7 S1pr1 Zfpm1 Dsp Actc1 Epor Ctnnb1 Angpt1 |
| Up | Muscle fiber development | 1.73 | 51 | 0.00033 | Myom1 Obsl1 Neb Myom2 Myom3 Mybpc2 Nrap Six1 Ttn Nebl Rcan1 Dner Flnc Tcap Myh11 Bmp4 Chrnb1 Lmod3 Klhl40 Klhl41 Xk Hdac4 Uchl1 Dysf Dmd Myof Cav2 Smo Actn3 Homer1 Cacna2d2 Cacnb4 Sgcd Ptcd2 Bin3 Cxadr Wnt10b Vegfa Afg3l2 Lox Shox2 Ppp3ca Sgcb Ryr1 Comp Fbxo22 Myh6 Zmpste24 Wfikkn2 Bcl2 Naca Gpx1 Myo18b |
| Up | Multicellular organismal movement | 1.80 | 43 | 0.00033 | Casq1 Tnnt2 Casq2 Dmpk Tnni2 Tnni3 Tnnt1 Tnnc1 Scn4a Tcap Mb Rps6kb1 Vti1a Chuk Gaa Atp2a2 Atp2a1 Myh14 Eef2 Chrnb1 Kcnj2 Xrcc1 Myh7 Cav3 Grcc10 Actn3 Homer1 Ascl1 Vps54 Atp8a2 Cln8 Map1a Mtor Itpr1 Synm Vps35 Comp Cacna1a Dmd Gigyf2 Hipk2 Bscl2 Slc8a3 Mylk2 Adrb2 Rcsd1 |
| Up | Positive regulation of muscle contraction | 1.79 | 27 | 0.00057 | Adra1a Edn1 Tbxa2r Chrm3 Kit Rhoa Kcnq1 Itga2 Srf Ccn2 Tacr2 Myocd Atp2a1 Cttn Ptgs2 Nppa Oxtr Gper1 Ptafr Abat Rnf207 Sphk1 Actn3 Ada Smtn Rgs2 Atp1a1 Npnt Lck Map2k1 Mylk2 Pawr Trpm4 Ptgs1 F2r |
| Up | Sarcoplasmic reticulum calcium ion transport | 1.75 | 25 | 0.0019 | Casq1 Trdn Fkbp1b Casq2 Dhrs7c Ank2 Ryr2 Nol3 Gsto1 Atp2a2 Camk2d Slc8a1 Ccr5 Tmem38b Tmem38a Pde4d Ryr1 Pln Chd7 Sln Zmpste24 Dmd Gstm7 Atp2a1 Hrc Cacna1c |
| Up | Pericardium development | 1.66 | 20 | 0.011 | Tbx5 Ccm2 Bmp7 Wnt5a Smad2 Bmp5 Smad3 Heg1 Sos1 Notch1 Dll4 Bmp2 Mecom Tbx20 Rpgrip1l Hand2 Setd2 Flrt3 Epor Rxra Vcam1 Pdpn |

**S-Table 1(b) :** Upregulated genes in Brn-3b KO aortas linked to metabolic or immune response pathways or circadian processes and organised by number of genes, with NES and p-value indicating significance.

| **Table 1b** | **Pathways** | **NES** | **nGenes** | **adj.Pval** | **Genes** |
| --- | --- | --- | --- | --- | --- |
| **Metabolic/Immune responses:** | | | | | |
| Up | Multicellular organismal movement | 1.80 | 43 | 0.00033 | Casq1 Tnnt2 Casq2 Dmpk Tnni2 Tnni3 Tnnt1 Tnnc1 Scn4a Tcap Mb Rps6kb1 Vti1a Chuk Gaa Atp2a2 Atp2a1 Myh14 Eef2 Chrnb1 Kcnj2 Xrcc1 Myh7 Cav3 Grcc10 Actn3 Homer1 Ascl1 Vps54 Atp8a2 Cln8 Map1a Mtor Itpr1 Synm Vps35 Comp Cacna1a Dmd Gigyf2 Hipk2 Bscl2 Slc8a3 Mylk2 Adrb2 Rcsd1 |
| Up | Rhythmic behaviour | 1.81 | 27 | 0.00033 | Btbd9 Ptgds Egr2 Ada Adora2a Srd5a1 Csnk1e Cst3 Per3 Star Adora1 Naglu Ncoa2 Pten Id2 Mta1 Chrnb2 Mtor Usp2 Egr1 Ciart Kcna2 Gpr157 Trp53 Kcnd2 Nlgn1 Casp1 Ahcy Alb Ptger4 Lepr |
| Up | Acute-phase response | 1.70 | 22 | 0.0049 | Il1b Ptgs2 Stat3 Hfe Cd163 Lbp Itih4 Ednrb Fn1 Tfr2 F8 Hp Orm1 Saa3 Cnr1 Orm2 Serpina1b Plscr1 Trpv1 Sigirr Tnfrsf11a Stat5b Ahsg Il1rn Ccl5 Ccr5 |
| Up | Corticosteroid receptor signalling pathway | 1.67 | 16 | 0.011 | Cry1 Clock Ppp5c Arid1a Ywhah Nr3c1 Jak2 Nedd4 Phb Cry2 Per1 Arntl Ptges3 Ncoa2 Bdnf Ntrk2 |
| Up | Animal organ maturation | 1.71 | 17 | 0.0081 | Phospho1 Igf1 Sema4d Bmp2 Mbtps2 Lep Actn3 Rhoa Aldh1a2 Gata3 Thbs3 Ret Ryr1 Dchs1 Fat4 Plxnb1 Ano6 Ltf |
| Up | Glucocorticoid receptor signalling pathway | 1.69 | 15 | 0.015 | Cry1 Clock Ppp5c Arid1a Ywhah Nr3c1 Nedd4 Phb Cry2 Per1 Arntl Ptges3 Ncoa2 Bdnf Ntrk2 |
| Up | Bone maturation | 1.74 | 15 | 0.0058 | Phospho1 Igf1 Sema4d Bmp2 Mbtps2 Lep Actn3 Rhoa Thbs3 Ryr1 Dchs1 Fat4 Plxnb1 Ano6 Ltf |

| **Table 1b** | **Pathways** | **NES** | **nGenes** | **adj.Pval** | **Genes** |
| --- | --- | --- | --- | --- | --- |
| **Circadian processes** | | | | | |
| Up | Circadian rhythm | 1.66 | 144 | 0.00033 | Ppp1cc Id3 Ppp1cb Id2 Nr1d1 Per1 Id4 Nr1d2 Fbxl3 Csnk1d Per3 Srrd Fbxl21 Ciart Ppp1ca Id1 Nrip1 Prokr1 Nfil3 Rai1 Btbd9 Ptgds Cry1 Ppara Creb1 Rorc Hdac1 Ppargc1a Clock Rora Rbm4b Pml Hnrnpu Mybbp1a Ep300 Arntl Cry2 Rbm4 Ndufa9 Hnrnpd Cdk4 Prox1 Hnrnpl Ada Cdk1 Sirt1 Egfr Adora2a Ddc Adcy1 Nampt Slc6a4 Srd5a1 Csnk1e Usp7 Adipoq Maged1 Btrc Huwe1 Ube3a Tyms Cst3 Fbxw7 Ezh2 Bhlhe40 Usp9x Nono F7 Star Mycbp2 Piwil2 Klf9 Cipc Siah2 Klf10 Timeless Tph1 Arntl2 Adora1 Thrap3 Spsb4 Jun Kdm2a Per2 Lep Dbp Impdh2 Crem Hnrnpr Top1 Jund Ncoa2 Igf1 Srebf1 Nos2 Fas Ngf Mttp Bhlhe41 Atf5 Atf4 Bdnf Lgr4 Ntrk2 Ntrk3 Trp53 Naglu Kmt2a Nr2f6 Relb Crtc1 Pten Fbxw11 Mapk9 Top2a Mta1 Opn4 Mapk8 Pspc1 Prkdc Gsk3b Dyrk1a Sik1 Ddb1 Tnfrsf11a Chrnb2 Prkaa2 Sfpq Mtor Gfpt1 Kdm5a Atg7 Kdm8 Usp2 Ccar2 Ogt Egr1 Suv39h1 Kcna2 Tardbp Sin3a Setx Phlpp1 Gpr157 Prkaa1 Kcnd2 Nlgn1 Prkcg Casp1 Ahcy Alb Dpyd Prf1 Cyp7b1 Ptger4 Prkg1 Mat2a Lepr Kdm5c Kdm5b |
| Up | Circadian regulation of gene expression | 1.77 | 46 | 0.00033 | Ppp1cb Per1 Per3 Ciart Nrip1 Rai1 Ppp1cc Ppara Hdac1 Ppargc1a Rora Rbm4b Pml Hnrnpu Ppp1ca Mybbp1a Arntl Sirt1 Maged1 Huwe1 Clock Bhlhe40 Mycbp2 Kdm2a Top1 Cry1 Cry2 Kmt2a Relb Ncoa2 Nampt Id2 Nr1d1 Mta1 Csnk1e Csnk1d Rorc Gfpt1 Kdm5a Bhlhe41 Kdm8 Usp2 Ogt Egr1 Atf4 Lgr4 Rbm4 |
| Up | Circadian behaviour | 1.75 | 26 | 0.00057 | Btbd9 Ptgds Ada Adora2a Srd5a1 Csnk1e Cst3 Per3 Star Adora1 Naglu Ncoa2 Pten Id2 Mta1 Chrnb2 Mtor Usp2 Egr1 Ciart Kcna2 Gpr157 Trp53 Kcnd2 Nlgn1 Casp1 Ahcy Alb Ptger4 Lepr |
| Up | Entrainment of circadian clock by photoperiod | 1.76 | 18 | 0.0029 | Ppp1cc Per1 Fbxl3 Per3 Fbxl21 Ppp1ca Rbm4b Pml Rbm4 Nr2f6 Crtc1 Ppp1cb Cry1 Id2 Mta1 Sik1 Bhlhe40 Usp2 Trp53 Cry2 |
| Up | Photoperiodism | 1.76 | 18 | 0.0029 | Ppp1cc Per1 Fbxl3 Per3 Fbxl21 Ppp1ca Rbm4b Pml Rbm4 Nr2f6 Crtc1 Ppp1cb Cry1 Id2 Mta1 Sik1 Bhlhe40 Usp2 Trp53 Cry2 |
| Up | Sleep | 1.75 | 16 | 0.0043 | Ptgds Dlat Ada Adora2a Fos Srd5a1 Slc29a1 Cst3 Per3 Star Htr2a Adora1 Oxtr Chrnb2 Kcna2 Btbd9 Casp1 Snap25 Alb Il18 Ptger4 |
