# Supplementary material for: Vascular dysfunction caused by loss of Brn-3b/POU4F2 transcription factor in aortic vascular smooth muscle cells is linked to deregulation of calcium signalling pathways
Source: Cell Death Dis. 2023 Nov 25;14(11):770. doi: 10.1038/s41419-023-06306-w (PMC10676411; doi:10.1038/s41419-023-06306-w)
Supplement: Supplementary file 4 — Supplementary Table 2 [file 41419_2023_6306_MOESM4_ESM.docx]

**Supplementary data: S-Table 2:** (a) List of GO biological processes associated with enriched genes within each cluster using data from 2 WT and 3 Brn-3b KO. Adjusted p-value and number of genes associated with different pathways are shown for each cluster.

| **Cluster** | **adj.Pval** | **nGenes** | **GO Biological Processes** |
| --- | --- | --- | --- |
| **A** | **6.6e-07** | **53** | **Ion transport** |
|  | 1.4e-06 | 31 | Metal ion transport |
|  | 1.7e-06 | 54 | Transmembrane transport |
|  | 3.4e-05 | 34 | Cation transport |
|  | 1.8e-04 | 16 | Potassium ion transport |
|  | 2.0e-03 | 82 | Transport |
|  | 2.0e-03 | 82 | Establishment of localization |
|  | 2.9e-03 | 4 | Heart process |
|  | 2.9e-03 | 4 | Heart contraction |
|  | 2.9e-03 | 17 | Cell-cell adhesion |
|  | 2.9e-03 | 4 | Regulation of heart contraction |
|  | 2.9e-03 | 19 | Monovalent inorganic cation transport |
|  | 4.6e-03 | 83 | Localization |
|  | 4.8e-03 | 10 | Calcium ion transport |
|  | 6.7e-03 | 6 | Regulation of system process |
| **B** | **6.0e-03** | **8** | **Cell activation** |
|  | **6.0e-03** | **22** | **Immune system process** |
|  | 6.0e-03 | 6 | Lymphocyte activation |
|  | 6.0e-03 | 22 | Regulation of biological quality |
|  | 6.0e-03 | 32 | Ion transport |
|  | 6.0e-03 | 17 | Microtubule-based process |
|  | 9.7e-03 | 63 | Localization |

| **Cluster** | **adj.Pval** | **nGenes** | **GO Biological Processes** |
| --- | --- | --- | --- |
| **C** | **1.7e-03** | **4** | **Muscle cell differentiation** |
|  | 1.7e-03 | 4 | Striated muscle cell differentiation |
|  | 2.9e-03 | 3 | Muscle cell development |
|  | 2.9e-03 | 3 | Striated muscle cell development |
|  | 2.9e-03 | 3 | Muscle tissue development |
|  | 2.9e-03 | 3 | Striated muscle tissue development |
|  | 8.5e-03 | 4 | Muscle structure development |
| **D** | **6.6e-07** | **31** | **Immune system process** |
|  | 7.9e-05 | 4 | Regulation of cytokine-mediated signaling pathway |
|  | 7.9e-05 | 4 | Regulation of response to cytokine stimulus |
|  | 1.5e-04 | 11 | Inflammatory response |
|  | 3.9e-04 | 22 | Immune response |
|  | 4.3e-04 | 21 | Defense response |
|  | 9.9e-04 | 21 | Response to chemical |
|  | 1.3e-03 | 18 | Response to external stimulus |
|  | 1.6e-03 | 8 | Response to cytokine |
|  | 3.8e-03 | 6 | Cytokine-mediated signaling pathway |
|  | 4.3e-03 | 11 | Regulation of immune system process |
|  | 7.0e-03 | 7 | Cellular response to cytokine stimulus |

| **Cluster** | **adj.Pval** | **Genes** | **GO Cellular component** |
| --- | --- | --- | --- |
| **A** | 1.1e-05 | 47 | Plasma membrane |
|  | 1.5e-05 | 47 | Cell periphery |
|  | 7.3e-05 | 25 | Integral component of plasma membrane |
|  | 9.2e-05 | 25 | Intrinsic component of plasma membrane |
|  | 2.0e-03 | 4 | Myofibril |
|  | 2.0e-03 | 4 | Sarcomere |
|  | 2.0e-03 | 4 | Contractile fiber |
|  | 2.0e-03 | 11 | Transmembrane transporter complex |
|  | 2.0e-03 | 11 | Transporter complex |
|  | 2.7e-03 | 10 | Ion channel complex |
|  | 2.7e-03 | 10 | Cation channel complex |
|  | 5.1e-03 | 3 | Troponin complex |
|  | 5.1e-03 | 3 | Striated muscle thin filament |
|  | 5.1e-03 | 6 | Voltage-gated potassium channel complex |
|  | 5.1e-03 | 6 | Potassium channel complex |
| **D** | 1.8e-03 | 38 | Extracellular region |

**S Table 2 (b)**
